# Pre-vaccination *Schistosoma mansoni* and hookworm infections are associated with altered vaccine immune responses: a longitudinal analysis among adolescents living in helminth-endemic islands of Lake Victoria, Uganda

**DOI:** 10.3389/fimmu.2024.1460183

**Published:** 2024-08-29

**Authors:** Agnes Natukunda, Ludoviko Zirimenya, Gyaviira Nkurunungi, Jacent Nassuuna, Ronald Nkangi, Alex Mutebe, Paul L. A. M. Corstjens, Govert J. van Dam, Alison M. Elliott, Emily L. Webb

**Affiliations:** ^1^ Immunomodulation and Vaccines Focus Area, Vaccine Research Theme, Medical Research Council/Uganda Virus Research Institute and London School of Hygiene and Tropical Medicine (MRC/UVRI and LSHTM) Uganda Research Unit, Entebbe, Uganda; ^2^ Medical Research Council (MRC) International Statistics and Epidemiology Group, Department of Infectious Disease Epidemiology, London School of Hygiene and Tropical Medicine, London, United Kingdom; ^3^ Department of Infection Biology, London School of Hygiene and Tropical Medicine, London, United Kingdom; ^4^ Department of Cell and Chemical Biology, Leiden University Medical Center, Leiden, Netherlands; ^5^ Department of Parasitology, Leiden University Medical Center, Leiden, Netherlands; ^6^ Department of Clinical Research, London School of Hygiene and Tropical Medicine, London, United Kingdom

**Keywords:** helminths, *Schistosoma mansoni*, hookworm, vaccines, immune responses, antibody responses

## Abstract

**Background:**

Variations in vaccine responses have been observed between populations. A role for helminth infections has been proposed due to their immunomodulatory properties. In a secondary analysis of data from a randomised trial assessing effects of anthelminthic treatment on vaccine responses, we examined associations between helminth infections at baseline prior to vaccine administration, and vaccine responses among adolescents (9-17 years) in Koome Islands, Lake Victoria, Uganda.

**Methods:**

Participants received BCG [week 0], yellow fever (YF-17D), oral typhoid (Ty21a), HPV-prime [week 4], and HPV-boost, tetanus/diphtheria [week 28]. Outcomes were BCG-specific interferon-γ ELISpot responses and antibody responses to yellow-fever-, typhoid-, HPV-, tetanus- and diphtheria-specific antigens measured at two time points post vaccination. *S. mansoni* infection was determined as positive if either the plasma Circulating Anodic Antigen (CAA) assay or stool PCR were positive. Hookworm and *Strongyloides* were determined by stool PCR. Linear mixed effects regression was used to assess associations.

**Results:**

Among 478 adolescents, 70% were *Schistosoma mansoni* (Sm) infected and 23% hookworm infected at baseline. *Sm* was associated with lower *Salmonella* Typhi O:LPS-specific IgG responses (adjusted geometric mean ratio (aGMR) 0.69 (0.57-0.83)), and hookworm with higher diphtheria-specific IgG (aGMR 1.16 (1.02, 1.31)) and lower HPV-16-specific IgG (aGMR 0.70 (0.55, 0.90)) post-vaccination. High *Sm* intensity was associated with lower BCG-specific interferon-γ and *S.* Typhi O:LPS-specific IgG.

**Conclusions:**

We found inverse associations between *Sm* and responses to two live vaccines, whereas hookworm was positively associated with diphtheria-specific IgG. These findings support the hypothesis that helminth infections can modulate vaccine responses, while also highlighting potential heterogeneity in the direction of these effects.

## Introduction

Vaccination is an essential tool in the prevention of infectious diseases. However, the efficacy and immunogenicity of some commonly used vaccines vary greatly by population, with low and middle income as well as rural populations often exhibiting impaired responses (1, 2). Several vaccines are affected, notably the Bacillus Calmette-Guérin (BCG) vaccine, whose efficacy against pulmonary tuberculosis varies around the world, with lower efficacy observed in regions near the equator (2). These settings are characterised by high exposure to immunomodulating infections, including parasites such as helminths (3). Alongside other factors, parasitic infections have been implicated as a potential cause of impaired efficacy and immunogeneicity.

Parasitic helminths infect both humans and animals. The most common types of helminths that infect humans are soil-transmitted helminths (3), which are transmitted via contaminated soil and can cause intestinal morbidity, anaemia and impaired growth, and schistosomes (4), which are a type of flatworm that are transmitted via freshwater snails and can cause a disease called schistosomiasis. The prevalence and distribution of these parasites varies by region and population (3, 5).

It is estimated that approximately 24% of the global population is infected with one or more soil-transmitted helminths, most commonly hookworm, *Ascaris*, and whipworm. Most of these infections are found in sub-Saharan Africa, the Americas, and East Asia (3). Schistosomiasis is estimated to affect nearly 240 million people globally (5). The majority of people affected by schistosomiasis due to *Schistosoma mansoni* infection live in Africa, the Middle East, the Caribbean, Brazil, Venezuela, or Suriname (5). The distribution of schistosomiasis is highly focal, being driven by proximity to fresh water. For example, a national representative survey conducted in Uganda in 2017 estimated the prevalence of schistosomiasis to be around 29% (6). However, within the country, the prevalence of helminths varies by region. In the islands of Lake Victoria, helminths, primarily *S. mansoni*, affect nearly 70% of the population (7). Despite the use of mass drug administration with praziquantel, the only widely-used treatment option for schistosomiasis, prevalence remains high often due to high reinfection rates.

One way in which helminths may impact vaccine responses is through modulation of immune responses. Helminths secrete a range of molecules that can modulate the activity of immune cells, including T cells, B cells, and macrophages (8). For example, helminths have been shown to alter the balance of T helper cell subsets, leading to a shift in Th2 and regulatory T cell responses (8). They also affect antigen presenting cells (APCs); for example, by driving an alternatively activated macrophage phenotype (9) or dendritic cell-mediated induction of regulatory responses (10). This may be important when encountering a new vaccine antigen thereby potentially affecting the ability of vaccines to elicit an effective immune response. Why some vaccine responses appear to be more affected than others is not fully known; however, helminths, for example, may modify the immune system’s reaction to live vaccines by modifying innate immune response profiles (11).

Reviews found that, in animal models, helminths generally impair priming and accelerate waning of vaccine responses (12, 13). Studies in humans have found more mixed results when investigating the effect of helminths on vaccine responses (12). For example, one study conducted in Ethiopia found that anthelminthic treatment improved IFN-γ and IL-12 cellular responses to purified protein derivative (PPD) post-BCG vaccination, while the untreated group had higher PPD-specific TGF-β responses, implying a role for helminth-induced immunosuppression in impaired BCG responses (14) However, another study conducted in Ecuador did not find a similar effect on responses to BCG (15). Our recent meta-analysis on the effect of helminths on vaccine responses found that generally helminths are associated with lower responses and that the effects are vaccine specific, and subject to a high level of heterogeneity (12).

The mixed findings from available studies suggest that the effect of helminths on vaccine responses may vary depending on the specific vaccine and the host immune response. In this secondary analysis of data from a randomised trial, we sought to investigate the association of helminth infections with vaccine immune responses to a panel of vaccines administered to Ugandan adolescents living in a helminth-endemic setting. The portfolio of licenced vaccines assessed comprised live and inert, oral, and parental, priming, and boosting vaccines.

## Materials and methods

### Study setting, design, participants, data

**Figure 1:**
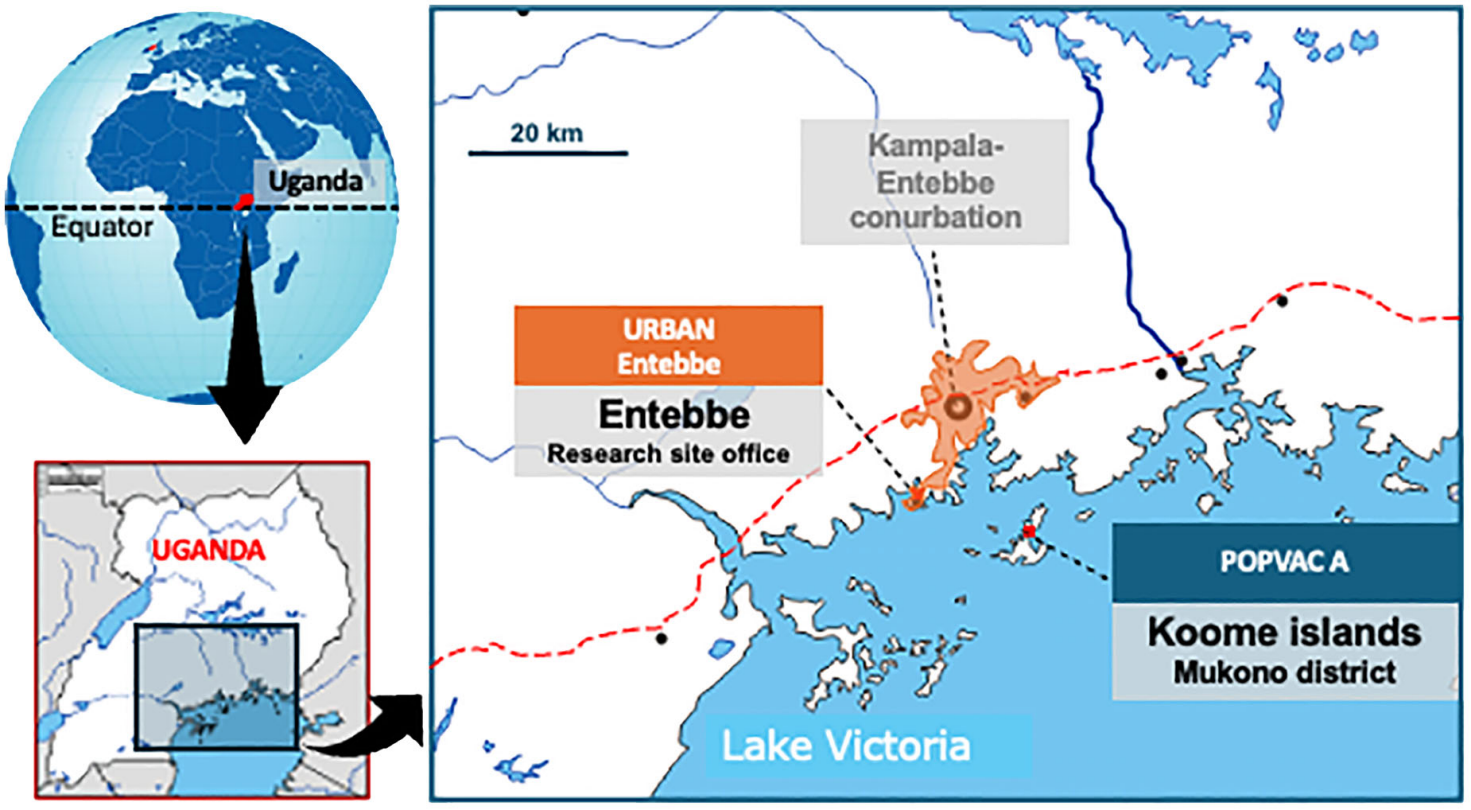
POPVAC A study site: The map of the study site shows Koome islands in Lake Victoria, where the study took place. These Islands are approximately 60 minutes from the nearest urban setting of Entebbe by a powered boat.

This was a secondary analysis of data from the longitudinal ‘POPVAC A’ trial. The trial assessed the effect of intensive treatment of schistosomiasis on vaccine responses (16, 17). It was conducted among adolescents (9-17 years) living in the schistosomiasis-endemic Koome islands of Lake Victoria, Uganda. The study setting is located approximately 60 minutes from the closest mainland by powered boat and comprises mainly fishing communities (**Figure 1**). Participants were recruited from eight selected primary schools in the islands.

**Figure 2:**
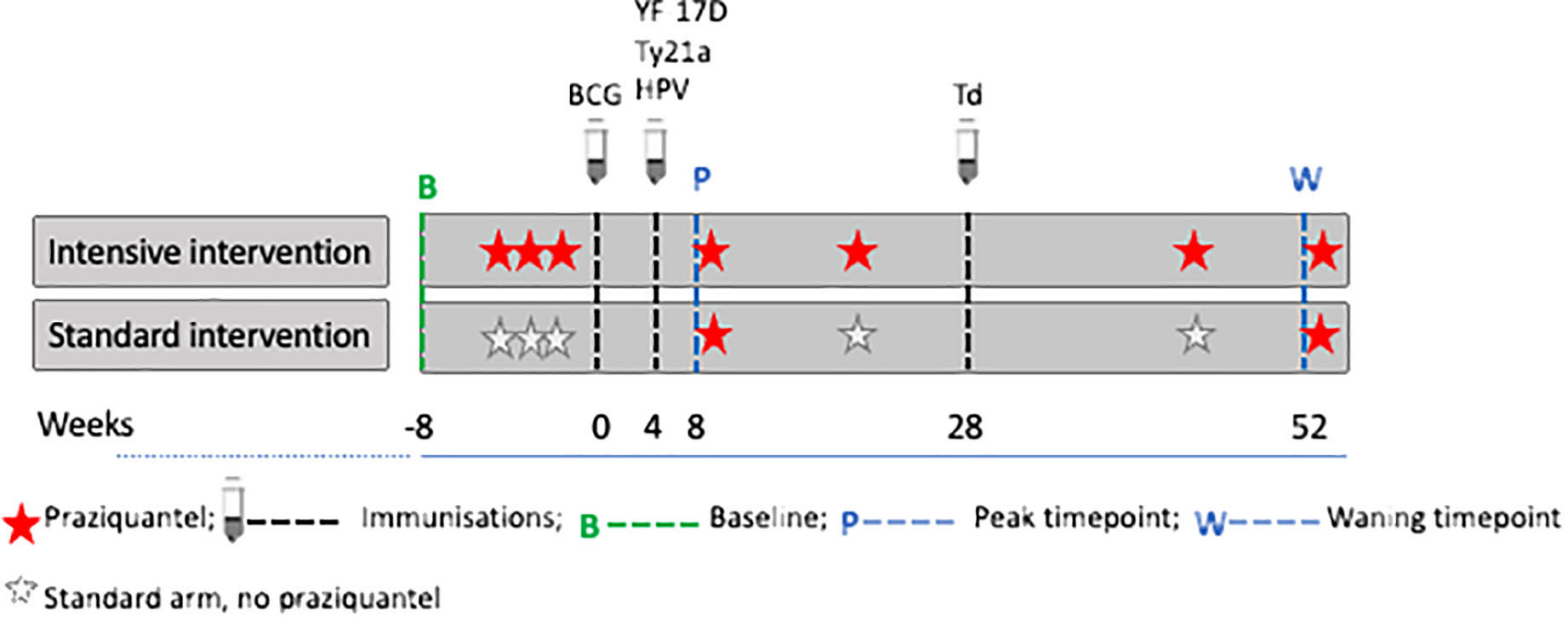
Praziquantel treatment, vaccine administration and response measurement time points. Figure adapted from (16). Peak timepoints were at 8 weeks post BCG and 4 weeks post yellow fever (YF- 17D), oral typhoid (Ty21a), human papilloma virus (HPV) and 24 weeks post tetanus/diphtheria (Td) vaccination. Waning timepoints were at 52 weeks post BCG and 48 weeks post YF- 17D, Ty21a, and HPV.

Socio-demographic characteristics of participants were collected using a questionnaire at baseline (six weeks prior to the first vaccination). For vaccinations, we selected a portfolio that would provide insights into the effects of both live and inert, oral and parenteral vaccines, while also being valuable to the age-group and communities under study. Six vaccines were administered: live parenteral BCG (Serum Institute of India, 0.1ml intradermally, right upper arm), live yellow fever (YF-17D, Sanofi Pasteur, France 0.5ml intramuscularly [IM], left upper arm), live oral typhoid (Ty21a, Vivotif, PaxVax, UK: three capsules, one per day taken every other day), virus-like particle Human Papillomavirus vaccine (HPV; Gardasil, Merck & Co, USA, 0.5ml IM, left upper arm) and toxoid vaccines (Tetanus/Diphtheria, Td, Serum Institute of India, 0.5ml IM, left upper arm), according to the schedule shown in **Figure 2**, i.e. BCG at week 0; YF-17D, Ty21a, and HPV at week 4; and Td at week 28. The trial enrolled 478 participants, half received intensive treatment for schistosomiasis (n=239) and the other half received standard treatment (n=239). Participants in the intensive arm received three doses of praziquantel (PZQ), 40mg/kg, at -6, -4 and -2 weeks, prior to the first vaccination, BCG at week 0, followed by PZQ at 8 weeks (after samples were taken for assessing vaccine responses at this time point) and then quarterly (weeks 20, 44 and 52) PZQ until the end of the 12-month follow-up. Participants in the standard arm received a dose of PZQ after week 8 and 52 samples had been taken; this is equivalent to standard of care which recommends annual treatment in schistosomiasis-endemic communities in Uganda.

### Outcomes and exposures

Except for Td, responses to vaccines for each participant were measured at three time points, i.e., pre-vaccination (at week 0, i.e. when BCG was administered); the second measurements were taken at 8 weeks post-BCG and 4 weeks post-YF, Ty21a, and HPV (peak timepoint); the third measurements were taken at 52 weeks post-BCG i.e. 48 weeks post-YF, Ty21a and HPV (waning timepoint). Responses to tetanus and diphtheria were measured at week 28 (pre-vaccination for Td) and at week 52 (24 weeks after Td vaccination). The treatment, vaccine administration and response measurement time points are shown in **Figure 2**.

Vaccine response measurements were BCG-specific interferon gamma (IFN-γ) ELISpot responses, YF-17D 50% and 90% neutralising antibody titres (assessed using plaque reduction neutralising reference tests, PRNTs), *S.* Typhi O-lipopolysaccharide (O:LPS)-specific IgG, HPV type 16 and type 18 L1 protein-specific IgG concentration, tetanus toxoid-specific IgG, and diphtheria toxoid-specific IgG. Assay details were previously reported (17) and are provided in the **Supplementary Information**.

Exposures were *S. mansoni*, hookworm, and *Strongyloides stercoralis* infections, assessed at baseline (before any intervention). *S. mansoni* infection was determined as positive if either the plasma Circulating Anodic Antigen (CAA) assay or stool PCR were positive. Hookworm and *Strongyloides* were determined by stool PCR. Helminth infection assessments were previously reported (17) and are further detailed below.

### Plasma detection of *Schistosoma* circulating anodic antigen

Baseline *S. mansoni* infection was determined retrospectively through measurement of plasma Circulating Anodic Antigen (CAA), using the up-converting phosphor lateral flow (UCP-LF) assay with a SCAA20 test format (30 pg/ml positivity threshold) (18). Briefly, Human negative serum (obtained from the Uganda blood bank, Nakasero) was spiked with a known concentration of CAA standard (100,000 pg/ml) and diluted up to eight standard points, with two negative controls. These were used to generate a standard curve to quantify the plasma sample CAA levels. Plasma and standards (50 µl) were extracted with an equal volume of 4% w/v trichloroacetic acid (TCA; Merck Life Science NV, the Netherlands), vortexed and incubated at ambient temperature and centrifuged. The resulting supernatant (20µl) was added to the wells containing 100 ng dry UCP particles (19) (400 nm Y2O2S:Yb3+, Er3+) coated with mouse monoclonal anti-CAA antibodies (20) hydrated with 100µl of high salt lateral flow buffer (HSLF: 200 mM Tris pH8, 270 mM NaCl, 0.5% (v/v) Tween-20, 1% (w/v) BSA). These were incubated for one hour at 37°C while shaking at 900 rpm. The CAA lateral flow strips (21) were labelled with the standard and sample identifications and then placed in the wells on the UCP plate. The samples and standards were allowed to flow and left to dry overnight. The strips were then analysed using the Labrox Upcon reader (Labrox Oy, Turku, Finland).

### Stool PCR detection of *Schistosoma mansoni*, *Necator americanus* and *Strongyloides stercoralis* DNA

Stool samples stored at -80^0^C in 95% molecular grade ethanol were retrieved and thawed at room temperature. Total DNA (and hence helminth DNA, if present) was extracted from the stool samples using the Fast DNA Spin Kit for Feces (catalogue number 116570200, MP Biomedicals Germany GmbH). The multiplex real-time PCR was adapted from existing procedures (22, 23). The following are the specific forward (F) and reverse (R) primers and TaqMan^®^ probes that were used to simultaneously detect DNA from three helminth species: *Necator americanus (*Na58F: 5’-CTGTTTGTCGAACGGTACTTGC-3’; Na158R: 5’- ATAACAGCGTGCACATGTTGC-3’; Nec-2-FAM (MGB): FAM-5’-CTGTACTACGCATTGTATAC-3’-XS; Nec-3-FAM (MGB): 5’-CTGTACTACGCATTGTATGT-3’), *Schistosoma mansoni* (Ssp48F: 5’-GGTCTAGATGACTTGATYGAGATGCT-3’; Ssp124R: 5’-TCCCGAGCGYGTATAATGTCATTA-3’; Ssp78T-RT: Texas Red-5’-TGGGTTGTGCTCGAGTCGTGGC-3’-BHQ2), *Strongyloides stercoralis* (Stro18S-1530F: 5’-GAATTCCAAGTAAACGTAAGTCATTAGC-3’; Stro18S-1630R: 5’-TGCCTCTGGATATTGCTCAGTTC-3’; Stro-4-TRBhq2-VIC- 5’-ACACACCGSCCGTCGCTGC-3’). Phocine herpes virus (PhHV) DNA, extracted from the Phocine herpes virus (kindly provided by Dr. Martin Schutten, Erasmus Medical Center, Rotterdam, the Netherlands), was included in the PCR master mix, thus distributed to all reaction wells as an internal control to check for PCR inhibition. The PhHV forward primer PhHV-267s (5’-GGGCGAATCACAGATTGAATC-3’), reverse primer PhHV-337as (5’-GCGGTTCCAAACGTACCAA-3’) and probe PhHV-305tq (Cy5-5’ TTTTTATGTGTCCGCCACCATCTGGATC-3’-BHQ2) were used for Phocin herpes virus DNA detection. A positive pool was included on the plate for every run as a test control. The positive pool was made up of a mixture of DNA from samples (from among the study samples) that were highly positive for *S. mansoni* and *N. americanus* on Kato-Katz, and for *S. stercoralis* by PCR (conducted on samples from a previous study). The amplification conditions were 15 minutes at 95°C, 50 cycles of 15 seconds at 95°C, 30s at 60°C and 30s at 72°C. DNA amplification, detection and data analysis were attained with the ABI 7500 Fast Real time machine and 7500 Fast systems software version 1.5.1.

### Statistical methods

The current analysis aimed to assess associations between baseline helminth infection status and responses to BCG, YF, HPV, Ty21a and Td, following vaccination with these vaccines. Baseline characteristics at enrolment prior to any interventions were summarised by *S. mansoni* and hookworm infection statuses and Chi-square tests (for categorical variables) and Wilcoxon rank sum tests (for continuous variables) were performed to examine if there were differences in the baseline characteristics between the groups.

A linear regression analysis was used to determine whether there was an association between baseline helminth infection status and vaccine responses at the peak and waning timepoints separately. Because vaccine responses had skewed distributions, they were log_10_ transformed for analysis. Geometric means and Geometric mean ratios (GMR) with 95% confidence intervals (CI) were obtained and presented. Both univariable and multivariable results, adjusted for confounders, are presented. Confounders were included in the multivariable models based on their plausible relationship with exposures and outcomes as identified in the literature. Multivariable models controlled for the following potential confounders: age, sex, body mass index, history of anthelminthic treatment, immunisation history, malaria infection and antibodies, the environment (village, town/city) where the participant lived at birth and five years and diet at baseline. Diet information was collected as the number of days in a week when fruits, vegetables, fish, and meat are consumed. For analysis, a diet score was calculated as the sum of days of consumption. The score ranged between 0 and 28, with a score of 28 representing daily consumption of the four food items every week i.e. greater variety of foods consumed.

For BCG, yellow fever, oral typhoid and HPV where responses after vaccinations were measured at the peak and waning timepoints, a linear mixed model with random intercepts was used to examine the associations of *S. mansoni* and hookworm infections with vaccine responses while controlling for confounders. In this analysis, an interaction between infection status and trial arm was explored. This was to determine whether associations between infections and vaccine responses differed depending on what treatment participants received after baseline infection status was determined. The analysis included responses at peak and waning time points. The use of a linear mixed model allowed us to account for dependencies in the data, arising from repeated measurements of the vaccine responses for each participant.

The analyses described above were repeated, but this time focusing on *S. mansoni* intensity levels as the main exposure rather than infection status. The intensity categories were based on tertiles for high, medium, and low intensity. This was done for *S. mansoni* only because the other helminths were less prevalent.

Stata 18.0 (College Station, Texas, USA) and R version 4.2.0 (R Foundation for Statistical Computing, Vienna, Austria) were used to analyse the data. For all analyses, a 5% significance level was used.

### Ethics approval

Ethics approval was granted by the Uganda Virus Research Institute Research Ethics Committee (references: GC/127/18/09/680, GC/127/19/05/664), the London School of Hygiene and Tropical Medicine Observational/Interventions Research Ethics Committee (reference: 16032), the Uganda National Council for Science and Technology (reference: HS2486) and the Uganda National Drug Authority (reference: CTA0093).

## Results

### Participants’ characteristics

The study enrolled 478 adolescents, of whom 335 (70.1%) were *S. mansoni* positive. Among 475 participants with baseline information on other helminth species (excluding 3 for not having a stool sample), 111 (23.4%) were *Necator americanus* (hookworm) positive and 38 (8.0%) were *Strongyloides stercoralis* positive. Baseline characteristics of all study participants, as well as categorised by helminth infection status, are presented in **Table 1**. The median age of participants at enrolment was 11 years (interquatile range (IQR) 10.0–13.0) with 57.7% being male. *S. mansoni*-positive participants were more likely to be born in a village setting, have a lower diet score, come from a household without a toilet and rely on lake or spring water for drinking rather than well or piped water. Hookworm-positive participants were more likely to be male and have a higher diet score. Other characteristics were similar between the helminth infection status groups.

**Table 1:** Baseline characteristics of participants.

	*S. mansoni* negative (n=143)	*S. mansoni* positive (n=335)	P value	Hookworm negative (n=364)	Hookworm positive (n=111)	P value	Total (n=478)
Characteristic							
Median age, years (IQR)	11 (10-13)	11 (10-13)	0.076	11 (10-13)	12 (10-13)	0.300	11 (10-13)
Sex, Male	73 (51.1%)	203 (60.6%)	0.053	199 (54.7%)	74 (66.7%)	**0.025**	276 (57.7%)
Median Body Mass Index (BMI) (IQR)	16.6 (15.8-17.9)	17.1 (16.0-18.3)	0.062	17.0 (16.0-18.2)	16.8 (15.8-18.2)	0.695	16.9 (15.9-18.2)
Received immunisation as a baby before school (mv 0,1) (mv 0,1) (mv 1)							
Yes	120 (83.9%)	269 (80.5%)	0.658	298 (81.9%)	88 (80.0%)	0.795	389 (81.5%)
No	10 (7.0%)	26 (7.8%)		26 (7.1%)	10 (9.1%)		36 (7.6%)
Don’t know	13 (9.1%)	39 (11.7%)		40 (11.0%)	12 (10.9%)		52 (10.9%)
Received immunisation since starting school (mv 0, 1) (mv 1,1) (mv 1)							
Yes	5 (3.5%)	21 (6.3%)	0.418	23 (6.3%)	3 (2.7%)	0.235	26 (5.5%)
No	128 (89.5%)	293 (88.0%)		316 (87.1%)	102 (92.7%)		421 (88.4%)
Don’t know	10 (7.0%)	19 (5.7%)		24 (6.6%)	5 (4.6%)		29 (6.1%)
Trial arm							
Intensive (Treated)	68 (47.5%)	171 (51.0%)	0.484	184 (50.6%)	54 (48.7%)	0.726	239 (50.0%)
Not treated (Standard)	75 (52.5%)	164 (49.0%)		180 (49.5%)	57 (51.4%)		239 (50.0%)
Reported worm treatment (past 12 months) (mv 27, 26) (mv 38, 15) (mv 53)	113 (90.4%)	264 (88.0%)	0.476	289 (88.7%)	86 (89.6%)	0.798	377 (88.7%)
Reported malaria treatment (past 12 months) (mv 2, 4) (mv 5, 1) (mv 6)	50 (35.5%)	129 (39.0%)	0.472	137 (38.2%)	41 (37.3%)	0.867	179 (37.9%)
Residence at birth (mv 4, 9) (mv 10,3) (mv 13)							
Village	120 (86.3%)	303 (92.9%)	**0.023**	320 (90.4%)	100 (92.6%)	0.487	423 (91.0%)
Town/city	19 (13.7%)	23 (7.1%)		34 (9.6%)	8 (7.4%)		42 (9.0%)
Residence between birth and age five years (mv 2, 6) (mv 6,2) (mv 8)							
Village	131 (92.9%)	311 (94.5%)	0.496	334 (93.3%)	105 (96.3%)	0.243	442 (94.0%)
Town/city	10 (7.1%)	18 (5.5%)		24 (6.7%)	4 (3.7%)		28 (6.0%)
Median diet score (days) (IQR) (mv 2, 7) (mv 6, 3) (mv 9)	10 (7-12)	8 (6-11)	**0.001**	9 (6-11)	9.5 (7-12)	**0.021**	9 (7-11)
Household owns a toilet (mv 1, 2) (mv 1,2) (mv 3)							
Yes	81 (57.0%)	143 (42.9%)	**0.005**	172 (47.4%)	51 (46.8%)	0.913	224 (47.2%)
No	61 (43.0%)	190 (57.1%)		191 (52.6%)	58 (53.2%)		251 (52.8%)
Source of drinking water (mv 1, 1) (mv 0,2) (mv 2)							
Well/Piped	89 (62.7%)	112 (33.5%)	**<0.001**	145 (39.8%)	54 (49.5%)	0.072	201 (42.2%)
Lake/spring	53 (37.3%)	222 (66.5%)		219 (60.2%)	55 (50.5%)		275 (57.8%)

*Schistosoma mansoni*: positivity was determine based on either CAA or PCR.

Mv Missing values are shown as the number missing for (S. mansoni negative, S. mansoni positive), (hookworm negative, hookworm positive), (overall).

IQR Interquartile range.

The bold values are p-values that reached statistical significance at P<=0.05.

### Pre-vaccination responses to vaccine antigens

Responses to selected vaccine antigens may be detected before adolescent vaccination (pre-vaccination) due to previous receipt of the same vaccines (for example in infancy for BCG, tetanus and diphtheria) or due to exposure to the target pathogen or cross-reactive organisms. Prior to vaccination during the study, *S. mansoni* positive (CAA/PCR) participants had lower BCG-specific IFN-γ responses (median 58.3 (IQR: 33.3-103.3) compared to 89.2 (IQR: 36.7-150.0), p = 0.018). Conversely, hookworm-positive participants had higher BCG-specific IFN-γ responses (91.7 (IQR:50.0-123.3) versus 61.7 (IQR: 31.7-105.0), p = 0.039) and higher HPV-16-specific IgG responses (4.0 (IQR: 2.8-6.9) versus 3.5 (IQR: 2.3 5.4), p = 0.048). *S. mansoni* and hookworm infected participants had comparable yellow fever PRNT_50_ and PRNT_90_ titres, *S.* Typhi O:LPS-specific IgG, HPV-18 IgG, tetanus toxoid-specific IgG, and diphtheria toxoid-specific IgG responses pre-vaccination compared to negative participants (**Table 2**).

**Table 2:** Pre-vaccination antigen-specific responses.

	*S. mansoni* negative (n=143)	*S. mansoni* positive (n=335)	P value	Hookworm negative (n=364)	Hookworm positive (n=111)	P value	Total (n=478)
Median pre-vaccination antigen-specific responses (IQR)							
BCG-specific IFN-γ (SFU per a million PBMCs) (mv 61, 132) (mv 153, 40) (mv 193)	89.2 (36.7-150.0)	58.3 (33.3-103.3)	**0.018**	61.7 (31.7-105.0)	91.7 (50.0-123.3)	**0.039**	66.7 (35-110)
Yellow fever PRNT_50_ titres (mv 12, 19) (mv 26, 5) (mv 31)	5.0 (5.0-5.0)	5.0 (5.0-5.0)	0.872	5.0 (5.0-5.0)	5.0 (5.0-5.0)	0.904	5.0 (5.0-5.0)
Yellow fever PRNT_90_ titres (mv 12, 19) (mv 26, 5) (mv 31)	5.0 (5.0-5.0)	5.0 (5.0-5.0)	0.837	5.0 (5.0-5.0)	5.0 (5.0-5.0)	0.904	5.0 (5.0-5.0)
*S.* Typhi O:LPS-specific IgG (mv 12, 19) (mv 26, 5) (mv 31)	112 (49.9-235.5)	124.9 (58.2-261.9)	0.507	117.6 (57.9-265.3)	129.9 (52.5-235.5)	0.921	120.9 (55.7-261.8)
HPV-16-specific IgG (mv 12, 19) (mv 26, 5) (mv 1) (mv 31)	3.8 (2.6-5.6)	3.5 (2.4-5.5)	0.427	3.5 (2.3-5.4)	4.0 (2.8-6.9)	0.048	3.6 (2.5-5.5)
HPV-18-specific IgG (mv 12, 19) (mv 26, 5) (mv 1) (mv 31)	59.2 (34.2-82.6)	63 (43.4-89.1)	0.119	62.8 (42.2-87.6)	60.2 (41.0-83.0)	0.313	62.4 (41.4-87.6)
Tetanus toxoid-specific IgG IgG (IU/ml)* (mv 41, 82) (mv 89, 33) (mv 123)	0.066 (0.033-0.122)	0.068 (0.035-0.147)	0.322	0.066 (0.033-0.140)	0.067 (0.036-0.138)	0.819	0.068 (0.035-0.140)
Diphtheria toxoid-specific IgG (IU/ml)* (mv 41, 82) (mv 89, 33) (mv 123)	0.058 (0.012-0.279)	0.045 (0.011-0.164)	0.230	0.044 (0.009-0.164)	0.071 (0.018-0.209)	**0.051**	0.050 (0.011-0.174)

*Schistosoma mansoni*: positivity was determine based on either CAA or PCR.

Mv Missing values are shown as the number missing for (S. mansoni negative, S. mansoni positive), (hookworm negative, hookworm positive), (overall).

IQR Interquartile range.

IFN-γ: interferon-γ; Yellow fever PRNT50: Yellow fever titres based on plaque reduction neutralizing reference tests at 50% neutralisation; PRNT90: Yellow fever Yellow fever titres based on plaque reduction neutralizing reference tests at 90% neutralisation; HPV-16: Human Papillomavirus type 16; HPV-18: Human Papillomavirus type 18.

The bold values are p-values that reached statistical significance at P<=0.05.

### Association between pre-vaccination characteristics and vaccine responses

**Figure 3:**
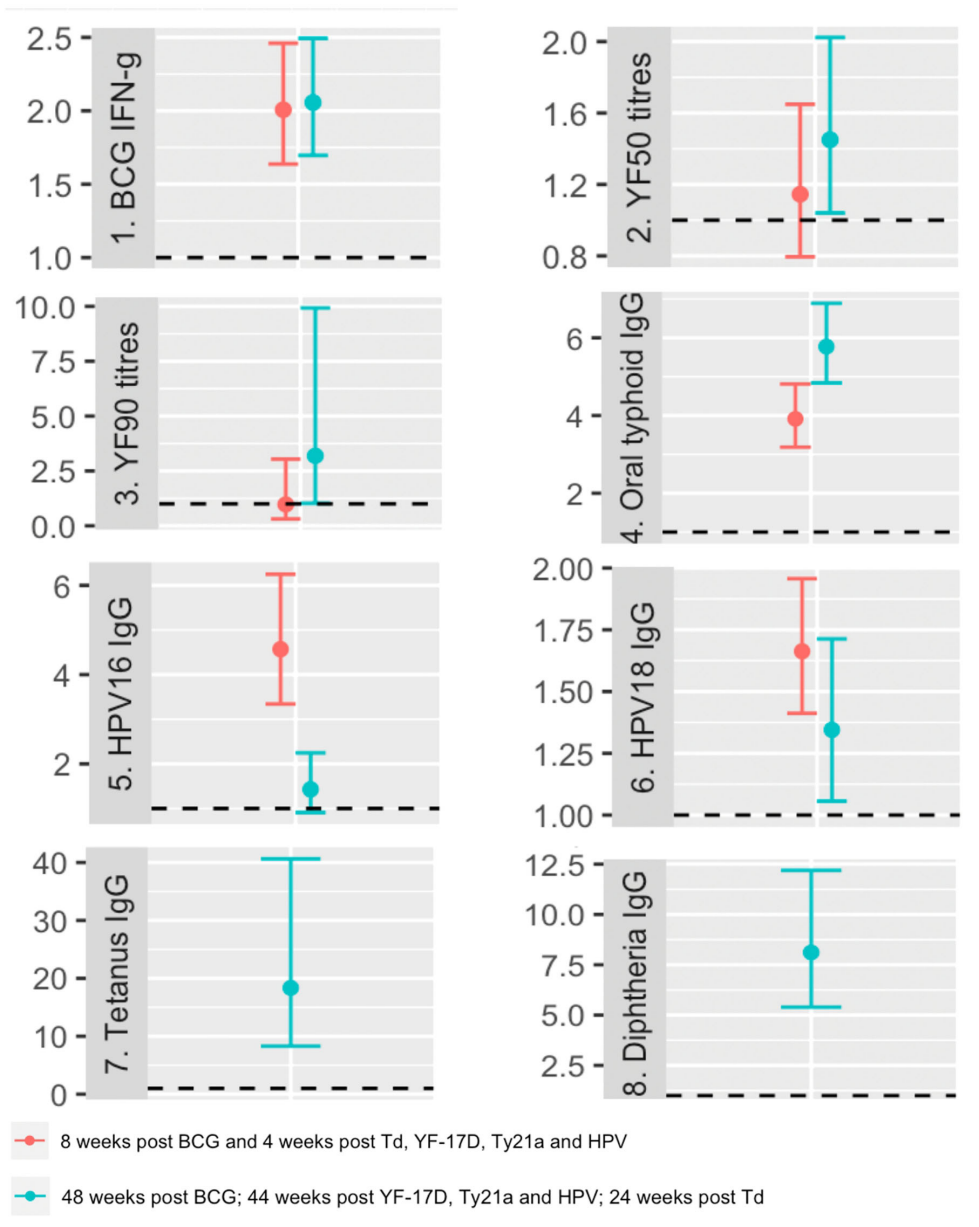
Crude associations between pre and post vaccination responses. The dots represent geometric mean ratios, and the bars 95% confidence intervals. The horizontal dashed line shows the null value of 1. IFN-g: interferon-γ; YF50: Yellow fever titres based on plaque reduction neutralizing reference tests at 50% neutralisation; YF90: Yellow fever titres based on plaque reduction neutralizing reference tests at 90% neutralisation; HPV-16: Human Papillomavirus type 16; HPV-18: Human Papillomavirus type 18.

**Supplementary Tables 1**, **2** present the results of analyses focusing on the crude associations between vaccine responses at each time point and various baseline characteristics, including exposures of interest (with focus on the most prevalent baseline helminths, *S. mansoni and* hookworm). Except for yellow fever titres four weeks after vaccination, post-vaccination responses to all vaccines investigated exhibited significant positive associations with their corresponding pre-vaccination responses (**Figure 3**).

At the peak timepoint, we observed that *S. mansoni* infection did not show a crude association with any vaccine responses. However, hookworm infection was inversely associated with HPV-16 responses, with a geometric mean ratio of 0.70 (95% CI 0.52, 0.93). Higher age was inversely associated with HPV 16 and 18-specific IgG responses, while displaying positive associations with tetanus and diphtheria toxoid-specific IgG responses (**Supplementary Table 1**).

We also observed sex related differences, with males exhibiting significantly lower HPV and tetanus toxoid-specific antibody responses compared to females. Participants who reported not receiving immunisation as babies showed lower tetanus toxoid-specific IgG responses, while those who reported receiving immunisation after starting school displayed lower yellow fever PRNT_50_ and PRNT_90_ titres and higher HPV responses. Participants in the intensive arm of the study had higher BCG-specific IFN-γ responses as reported elsewhere (**Supplementary Table 1**) (17).

Interestingly, participants who reported treatment of worm infection within 12 months prior to enrolment exhibited higher diphtheria toxoid-specific IgG responses. Current malaria infection and higher malaria-specific antibodies were associated with lower HPV responses. Moreover, participants from households that reported toilet ownership had higher BCG-specific IFN-γ, yellow fever PRNT_50_ and PRNT_90_ responses. Similarly, individuals whose drinking water source was from a well or piped source demonstrated higher BCG-specific IFN-γ responses but lower *S.* Typhi O:LPS-specific IgG responses. Lastly, a more varied diet was associated with higher BCG-specific IFN-γ responses (**Supplementary Table 1**).

At the waning timepoint, *S. mansoni* infection at baseline was inversely associated with *S.* Typhi O:LPS-specific IgG, while hookworm infection was inversely associated with tetanus toxoid-specific IgG. There were lower BCG-specific IFN-γ and yellow fever PRNT_50_ responses among participants with heavy *S. mansoni* infection compared to uninfected participants. Participants with medium infection intensity had lower HPV-18 responses compared to the uninfected participants. Males had significantly lower yellow fever and HPV titres. A higher body mass index was crudely associated with higher *S.* Typhi O:LPS-specific IgG responses. Participants who reported not being immunised as babies had significantly higher yellow fever PRNT_50_ and PRNT_90_ titres and lower HPV 16 responses (**Supplementary Table 2**). Plots of individual vaccine response profiles at each time point (pre-vaccination (week 0) and post vaccination (weeks 8 and 52)) by *S. mansoni* infection status and by *S. mansoni* intensity at baseline are shown in **Supplementary Figures S1**, **S2**.

**Figure 4:**
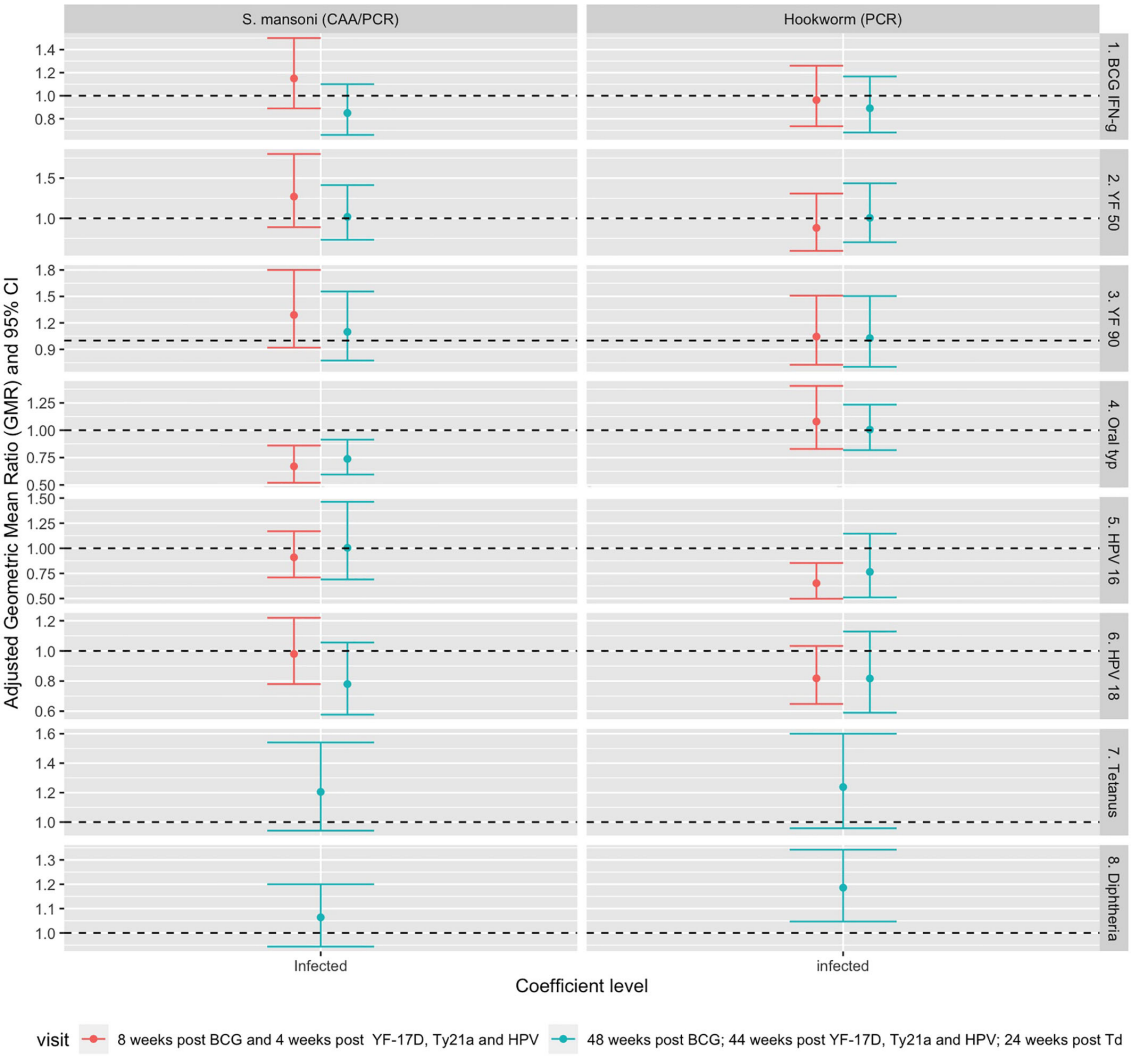
Adjusted association between helminth infections and vaccine responses. Shown are geometric mean rations (dots) and 95% CI (bars). The horizontal dashed line shows the null value of 1. S. mansoni: *Schistosoma mansoni*; CAA, circulating anodic antigen; PCR, Polymerase Chain Reaction; IFN-g, interferon-γ; YF50, Yellow fever titres based on plaque reduction neutralizing reference tests at 50% neutralisation; YF90, Yellow fever titres based on plaque reduction neutralizing reference tests at 90% neutralisation; HPV-16, Human Papillomavirus type 16; HPV-18, Human Papillomavirus type 18.

**Figure 4** shows associations between helminth infection and vaccine responses after adjusting for confounders. The analysis was first conducted separately for responses at each timepoint. *S. mansoni* infection at baseline was associated with lower *S.* Typhi O:LPS-specific IgG responses four weeks (aGMR 0.67 (95% CI 0.52, 0.86)) and 48 weeks (aGMR 0.74 (0.60-0.92)) after oral typhoid vaccination. Infection with hookworm at baseline was associated with lower HPV-16 responses (aGMR 0.66 (0.51, 0.86)) 44 weeks after vaccination and higher diphtheria-specific IgG responses 24 weeks after vaccination (aGMR 1.157 (1.02-1.31)). Detailed adjusted associations of helminth infection status and *S. mansoni* intensity with vaccine responses are shown in **Supplementary Tables 3**, **4**.

Finally, to combine vaccine response data from multiple time points into a single analysis, a linear mixed model was fitted to the data to account for correlation between responses of each individual at various timepoints. This analysis was done for responses that had measurements at two post-vaccination timepoints i.e. BCG-specific IFN-γ, yellow fever PRNT_50_, yellow fever PRNT_90_, *S.* Typhi O:LPS-specific IgG and HPV. The results adjusted for confounders are presented in **Table 3**. In this analysis, we investigated the interaction between infection status and trial arm to see if the association between *S. mansoni and* hookworm infection and vaccine responses differed by the study trial arm. There was no significant interaction between infection status and trial arm for any of the vaccine responses. *S mansoni* infection was inversely associated with *S.* Typhi O:LPS-specific IgG responses (aGMR 0.69 (0.57- 0.83)) whereas hookworm infection was inversely associated with HPV-16 IgG responses (aGMR 0.702 (0.549, 0.899)). **Supplementary Figures S3**, **S4** show the predicted responses by *S mansoni* and hookworm infections.

**Table 3:** Adjusted associations between helminth infections and vaccine responses for weeks 8 and 52 timepoints.

	*S. mansoni*		Hookworm	
	Adjusted GMR (95% CI)	P value	Adjusted GMR (95% CI)	P value
Infection status				
BCG-specific IFN-γ				
*Infection* (positive versus negative)	0.97(0.79, 1.19)	0.741	0.90(0.73, 1.11)	0.328
Yellow fever PRNT_50_ titres				
*Infection* (positive versus negative)	1.14(0.90- 1.45)	0.282	1.016(0.78, 1.32)	0.905
Yellow fever PRNT9_0_ titres				
*Infection* (positive versus negative)	1.19(0.93, 1.51)	0.163	1.05(0.81, 1.37)	0.707
*S. Typhi* O:LPS-specific IgG				
*Infection* (positive versus negative)	0.69(0.57, 0.83)	<0.001	1.12(0.92, 1.37)	0.267
HPV-16-specific IgG				
*Infection* (positive versus negative)	0.94(0.75, 1.19)	0.602	0.70(0.55, 0.90)	0.005
HPV-18-specific IgG				
*Infection* (positive versus negative)	0.88(0.73, 1.07)	0.211	0.82(0.67, 1.00)	0.054

Using the same modelling approach, we found a significant interaction between *S. mansoni* infection intensity and trial arm. *S. mansoni* infection intensity was associated with responses to BCG and oral typhoid with high intensity being associated with lower responses to BCG among standard arm participants only (aGMR 0.65 (0.46-0.91)) and high and medium *S. mansoni* infection intensities being associated with lower response to oral typhoid compared to *S. mansoni* negatives (aGMR 0.74 (0.59-0.94)) and (aGMR 0.70 (0.55-0.88)) respectively. Detailed results of associations of *S. mansoni* infection intensity and vaccine responses are presented in **Supplementary Table 5**.

## Discussion

We conducted an observational analysis to evaluate associations between baseline helminth infections and subsequent study-administered vaccine responses among Ugandan adolescents living in Koome Islands of Lake Victoria. In these fishing communities, where helminth infections, particularly *S. mansoni*, are highly prevalent, *S. mansoni* infection prior to vaccination was associated with lower *S.* Typhi O:LPS-specific IgG responses post vaccination. On the other hand, hookworm infection was associated with higher diphtheria toxoid-specific IgG responses and lower HPV-16 responses post vaccination. We also found that higher *S. mansoni* infection intensity was associated with lower BCG-specific IFN-γ and *S.* Typhi O:LPS-specific IgG responses. There was no significant association observed between helminth infections and responses to tetanus or yellow fever vaccines. These findings partly support the hypothesis that helminth infections may impair vaccine responses, although these effects were not consistent across all vaccines. In our study, negative effects were observed mainly for responses to BCG and oral typhoid vaccines both of which are live attenuated bacterial vaccines.

In the parent trial, we observed a significant reduction in schistosomiasis infection intensity following intensive treatment with praziquantel. Intensive treatment showed evidence of improved BCG-specific cellular response and no statistically significant improvement in the response to oral typhoid or responses to other studied vaccines (17). In the current observational analysis, we found an association between high *S. mansoni* infection intensity and lower BCG-specific IFN-γ responses in individuals who had not received treatment prior to vaccination. These findings are in line with a review that showed generally lower BCG-specific IFN-γ responses among helminth infected individuals (12).

We found that hookworm infection was associated with higher diphtheria-specific IgG and somewhat higher tetanus toxoid-specific IgG responses, consistent with findings from other studies that some worms can boost responses to some vaccines. Other studies that have shown this boosting effect include one for a malaria vaccine candidate, which found that hookworm and malaria coinfection resulted in higher IgG responses to the GMZ2 malaria candidate vaccine antigen and that hookworm treatment resulted in lower IgG levels (24). Adjuvant effects of helminths have also been observed for *Onchocerca volvulus* in mice (25) and human (26) studies. Interestingly, these effects have also been observed in studies investigating the effect of maternal helminth infection on vaccines given routinely in infancy. These boosting effects have been observed for orally administered polio and rotavirus vaccines, with a higher vaccine-specific response level observed in children born to helminth infected compared to uninfected mothers (27).

The lack of association between helminths, especially *S. mansoni* infection, and toxoid vaccines in our study was not unexpected, given that our recent meta-analysis of data from several studies found no association between responses to tetanus toxoid and helminth infection status prior to or at the time of vaccination (12). However, Riner and colleagues showed that among people who needed an immediate tetanus toxoid boost based on their baseline titres being less than 0.01 mIU/ml, those infected with *S. mansoni* had significantly lower tetanus responses than negative controls at six and eight months after the boost. In our study, we explored the association between helminths and vaccine responses in this subgroup of people for both tetanus and diphtheria but found no evidence of association.

Our research had some limitations. There was significant missing data for some exposures, such as prior treatment for worms and information on this, and other variables such as vaccinations received in infancy, were based on self-report and could be subject to measurement error. The study setting is highly endemic for *S. mansoni* and thus among participants who were negative at baseline, infections and reinfections may occur at short intervals, this might have inhibited us from seeing the full effect of *Sm* infection on vaccine responses and might not be typical of what happens in other settings. Also, as demonstrated elsewhere, the CAA test format (SCAA20) used to assess *S. mansoni* infection in our study was not the format with ultimate (single worm) sensitivity (28), implying that some individuals who were categorised as negative could carry a low worm burden infection. Following this observation, we investigated the association between *S. mansoni* intensity and vaccine responses, and the results supported what was observed for *S. mansoni* infection status and oral typhoid responses. Finally, given its low prevalence at baseline, *Strongyloides* was not included in the association analysis.

Overall, helminths have a complex effect on vaccine responses in humans. Certain species can impair the immunogenicity of some vaccinations, while others may have a neutral or positive effect (12). Our data show that being infected with *S. mansoni* prior to vaccination jeopardizes responses to some vaccines. The vaccines we tested generally produce robust protection, and a reduction in responses due to infection may not necessarily translate into individuals being unprotected. However, for some vaccines where benefits are more marginal, or for new vaccines, the reduction in responses among those infected or with heavy infection may be of public health concern. Although further research is needed to increase our understanding of the mechanisms behind these effects and their implications for public health, helminths can have both positive and negative effects on the effectiveness of certain vaccinations.

It is recommended that optimal vaccine response monitoring take place in helminth endemic settings, particularly for vaccines that have been shown to be negatively affected by helminths, allowing for potential boosting. Additionally, vaccine manufacturers should consider assessing the effect of helminths on candidate vaccines in early phase trials, especially for vaccines designed to be used in regions where helminths are endemic.

Further research should be conducted to investigate the mechanisms underlying the lower oral typhoid responses among those infected with *S. mansoni* as well as the potential boosting effects of hookworm infection on diphtheria toxoid-specific IgG responses. This will inform strategies to optimise vaccine effectiveness in communities at high risk of illness and death from infectious diseases.

## Data Availability

The de-identified individual participant data that underlie the results reported in this article are stored in a non-publicly available repository (LSHTM Data Compass), together with a data dictionary. Data are available on request via https://doi.org/10.17037/DATA.00003862. Researchers who would like to access the data may submit a request through LSHTM Data Compass, detailing the data requested, the intended use for the data, and evidence of relevant experience and other information to support the request. The request will be reviewed by the Principal Investigator in consultation with the MRC/UVRI and LSHTM data management committee, with oversight from the UVRI and LSHTM ethics committees. In line with the MRC policy on Data Sharing, there will have to be a good reason for turning down a request. Patient Information Sheets and consent forms specifically referenced making anonymised data available and this has been approved by the relevant ethics committees. Researchers given access to the data will sign data sharing agreements which will restrict the use to answering pre-specified research questions.
