# Supplementary material for: Pre-vaccination Schistosoma mansoni and hookworm infections are associated with altered vaccine immune responses: a longitudinal analysis among adolescents living in helminth-endemic islands of Lake Victoria, Uganda
Source: Front Immunol. 2024 Aug 29;15:1460183. doi: 10.3389/fimmu.2024.1460183 (PMC11390495; doi:10.3389/fimmu.2024.1460183)
Supplement: Supplementary file 1 [file DataSheet1.docx]

# **Supplementary information**

*Supplementary tables*

Table 1: Crude associations of pre-vaccination characteristics with vaccine responses at the first post-vaccination timepoint (8 weeks post BCG, 4 weeks post yellow fever, oral typhoid and HPV vaccinations and 24 weeks post Tetanus and diphtheria vaccination).

|  | **BCG-specific IFN-γ** | | **Yellow fever PRNT_50_ titres** | | **Yellow fever PRNT_90_ titres** | | ***S. Typhi* O:LPS-specific IgG** | | **HPV-16-specific IgG** | | **HPV-18-specific IgG** | | **Tetanus toxoid-specific IgG** | | **Diphtheria toxoid-specific IgG** | |
| --- | --- | --- | --- | --- | --- | --- | --- | --- | --- | --- | --- | --- | --- | --- | --- | --- |
| **Characteristic** | **GMR (95% CI)** | **P value** | **GMR (95% CI)** | **P value** | **GMR (95% CI)** | **P value** | **GMR (95% CI)** | **P value** | **GMR (95% CI)** | **P value** | **GMR (95% CI)** | **P value** | **GMR (95% CI)** | **P value** | **GMR (95% CI)** | **P value** |
| **Age, years** | 1.03  (0.98, 1.08) | 0.272 | 0.97  (0.90, 1.05) | 0.513 | 0.96  (0.89, 1.04) | 0.289 | 1.01  (0.95, 1.08) | 0.744 | 0.90  (0.84, 0.96) | 0.002 | 0.93  (0.88, 0.98) | 0.012 | 1.09  (1.02, 1.15) | 0.008 | 1.03  (1.00, 1.07) | 0.032 |
| **Sex (Male)** | 1.04  (0.87, 1.24) | 0.687 | 0.95  (0.73, 1.25) | 0.715 | 0.98  (0.75, 1.26) | 0.848 | 1.01  (0.80, 1.28) | 0.921 | 0.47  (0.37, 0.58) | <0.001 | 0.62  (0.51, 0.74) | <0.001 | 1.24  (1.00, 1.53) | 0.047 | 1.01  (0.91, 1.13) | 0.844 |
| **Body Mass Index (BMI)** | 1.03  (0.98, 1.07) | 0.258 | 1.04  (0.97, 1.12) | 0.278 | 1.03  (0.96, 1.10) | 0.435 | 1.04  (0.98, 1.11) | 0.205 | 0.95  (0.89, 1.01) | 0.077 | 0.95  (0.91, 1.00) | 0.064 | 1.04  (0.98, 1.10) | 0.164 | 1.02  (0.99, 1.05) | 0.321 |
| **Received immunisation as a baby before school** | | | | | | | | | | | | | | | | |
| No | 0.71  (0.52, 0.97) | 0.067 | 1.62  (0.99, 2.64) | 0.142 | 1.26  (0.79, 2.01) | 0.628 | 0.85  (0.55, 1.32) | 0.199 | 0.75  (0.49, 1.16) | 0.346 | 0.82  (0.58, 1.17) | 0.044 | 0.59  (0.40, 0.87) | 0.029 | 0.96  (0.79, 1.18) | 0.809 |
| Don’t know | 0.86  (0.65- 1.14) |  | 0.97  (0.64, 1.47) |  | 1.02  (0.69, 1.52) |  | 1.33  (0.93, 1.91) |  | 0.86  (0.59, 1.24) |  | 0.70  (0.52, 0.94) |  | 0.89  (0.64, 1.23) |  | 0.95  (0.81, 1.13) |  |
| Yes | Ref. |  | Ref. |  | Ref. |  | Ref. |  | Ref. |  | Ref. |  | Ref. |  | Ref. |  |
| **Received immunisation since starting school** | | | | | | | | | | | | | | | | |
| Yes | 0.80  (0.55, 1.16) | 0.445 | 0.48  (0.28, 0.81) | 0.007 | 0.46  (0.278, 0.77) | 0.006 | 1.23  (0.78, 1.96) | 0.376 | 3.34  (2.11, 5.28) | <0.001 | 3.10  (2.14, 4.48) | <0.001 | 0.89  (0.57, 1.37) | 0.755 | 0.90  (0.72, 1.11) | 0.469 |
| Don’t know | 0.90  (0.64, 1.27) |  | 0.61  (0.36, 1.04) |  | 0.70  (0.42, 1.16) |  | 1.32  (0.82, 2.14) |  | 0.85  (0.54, 1.34) |  | 0.88  (0.61, 1.27) |  | 0.88  (0.56, 1.39) |  | 0.91  (0.72, 1.15) |  |
| No | Ref. |  | Ref. |  | Ref. |  | Ref. |  | Ref. |  | Ref. |  | Ref. |  | Ref. |  |
| **Current Schistosoma treatment (trial arm)** | | | | | | | | | | | | | | | | |
| Treated | 1.20  (1.01, 1.43) | 0.038 | 0.97  (0.74, 1.26) | 0.807 | 0.98  (0.76, 1.27) | 0.887 | 1.11  (0.88, 1.40) | 0.367 | 0.82  (0.65, 1.03) | 0.091 | 0.93  (0.77, 1.12) | 0.436 | 1.16  (0.95, 1.43) | 0.149 | 0.97  (0.87, 1.07) | 0.508 |
| Untreated | Ref. |  | Ref. |  | Ref. |  | Ref. |  | Ref. |  | Ref. |  | Ref. |  | Ref. |  |
| **Reported treatment for worms in 12 months before enrolment** | | | | | | | | | | | | | | | | |
| Yes | 1.22  (0.91, 1.63) | 0.188 | 1.50  (0.96, 2.33) | 0.074 | 1.34  (0.88, 2.04) | 0.169 | 0.77  (0.523, 1.133) | 0.184 | 1.08  (0.74, 1.58) | 0.689 | 1.19  (0.87, 1.61) | 0.273 | 1.23  (0.87, 1.73) | 0.234 | 1.23  (1.03, 1.47) | 0.022 |
| No | Ref. |  | Ref. |  | Ref. |  | Ref. |  | Ref. |  | Ref. |  | Ref. |  | Ref. |  |
| Malaria infection status PCR | 1.04  (0.82, 1.30) | 0.772 | 0.59  (0.41, 0.84) | 0.003 | 0.63  (0.45, 0.89) | 0.009 | 0.81  (0.59, 1.11) | 0.183 | 1.03  (0.751, 1.416) | 0.850 | 0.96  (0.74, 1.24) | 0.740 | 1.14  (0.87, 1.49) | 0.359 | 0.94  (0.82, 1.07) | 0.331 |
| **Reported treatment for malaria in 12 months before enrolment** | | | | | | | | | | | | | | | | |
| Yes | 1.13  (0.94, 1.35) | 0.200 | 0.87  (0.66, 1.15) | 0.325 | 0.87  (0.67, 1.12) | 0.278 | 0.92  (0.73, 1.17) | 0.493 | 1.12  (0.88, 1.42) | 0.370 | 1.21  (0.99, 1.47) | 0.064 | 0.82  (0.66, 1.02) | 0.073 | 0.99  (0.89, 1.10) | 0.812 |
| No | Ref. |  | Ref. |  | Ref. |  | Ref. |  | Ref. |  | Ref. |  | Ref. |  | Ref. |  |
| **Malaria specific antibodies (PfAMA1)** | 1.07  (0.96, 1.19) | 0.252 | 0.91  (0.77, 1.07) | 0.266 | 0.93  (0.80, 1.09) | 0.373 | 0.95  (0.82, 1.09) | 0.462 | 0.92  (0.80, 1.06) | 0.261 | 0.94  (0.84, 1.06) | 0.329 | 1.06  (0.94, 1.20) | 0.348 | 1.07  (1.00, 1.13) | 0.051 |
| **Malaria specific antibodies (PfMSP2)** | 1.04  (0.80, 1.33) | 0.784 | 0.64  (0.435, 0.939) | 0.023 | 0.67  (0.47, 0.97) | 0.035 | 1.18  (0.85, 1.65) | 0.321 | 1.22  (0.87, 1.71) | 0.252 | 1.23  (0.94, 1.63) | 0.137 | 1.09  (0.81, 1.48) | 0.561 | 1.17  (1.00, 1.36) | 0.049 |
| **Own toilet** | | | | | | | | | | | | | | | | |
| Yes | 1.22  (1.02, 1.45) | 0.029 | 1.45  (1.11, 1.89) | 0.007 | 1.44  (1.11, 1.85) | 0.005 | 0.82  (0.65, 1.03) | 0.086 | 1.02  (0.81, 1.30) | 0.848 | 1.05  (0.86, 1.27) | 0.638 | 1.02  (0.83, 1.26) | 0.825 | 1.09  (0.98, 1.21) | 0.114 |
| No | Ref. |  | Ref. |  | Ref. |  | Ref. |  | Ref. |  | Ref. |  | Ref. |  | Ref. |  |
| **Source of drinking water** | | | | | | | | | | | | | | | | |
| Well/piped water | 1.23  (1.04, 1.47) | 0.019 | 1.01  (0.77, 1.32) | 0.964 | 1.07  (0.82, 1.38) | 0.627 | 0.76  (0.60, 0.96) | 0.019 | 1.09  (0.86, 1.39) | 0.474 | 0.92  (0.76, 1.12) |  | 1.00  (0.81, 1.24) | 0.973 | 1.09  (0.98, 1.21) | 0.130 |
| Lake/spring | Ref. |  | Ref. |  | Ref. |  | Ref. |  | Ref. |  | Ref. |  | Ref. |  | Ref. |  |
| **Residence of participant at birth** | | | | | | | | | | | | | | | | |
| Village | 1.08  (0.78, 1.49) | 0.651 | 1.47  (0.88, 2.45) | 0.141 | 1.41  (0.87, 2.30) | 0.167 | 0.79  (0.50, 1.22) | 0.283 | 1.15  (0.74, 1.79) | 0.534 | 0.93  (0.64, 1.33) | 0.680 | 0.90  (0.60, 1.35) | 0.596 | 0.88  (0.72, 1.08) | 0.228 |
| Town or city | Ref. |  | Ref. |  | Ref. |  | Ref. |  | Ref. |  | Ref. |  | Ref. |  | Ref. |  |
| **Residence of participant between birth and age five years** | | | | | | | | | | | | | | | | |
| Village | 0.98  (0.66, 1.45) | 0.924 | 1.81  (0.98, 3.32) | 0.057 | 1.70  (0.96, 3.04) | 0.071 | 0.69  (0.41, 1.16) | 0.160 | 1.71  (1.01, 2.91) | 0.047 | 1.11  (0.72, 1.72) | 0.635 | 0.79  (0.478, 1.31) | 0.357 | 0.96  (0.75, 1.23) | 0.740 |
| Town or city | Ref. |  | Ref. |  | Ref. |  | Ref. |  | Ref. |  | Ref. |  | Ref. |  | Ref. |  |
| **Diet score** | 1.02  (1.0, 1.05) | 0.041 | 1.01  (0.98, 1.05) | 0.500 | 1.01  (0.97, 1.04) | 0.647 | 1.01  (0.98, 1.05) | 0.411 | 0.99  (0.96, 1.02) | 0.509 | 0.98  (0.95, 1.01) | 0.131 | 0.98  (0.96, 1.01) | 0.281 | 1.01  (0.99, 1.02) | 0.539 |
|  |  |  |  |  |  |  |  |  |  |  |  |  |  |  |  |  |
| **Pre-vaccination antigen-specific responses** | | | | | | | | | | | | | | | | |
| Responses | 2.01  (1.64, 2.46) | <0.001 | 1.15  (0.80, 1.65) | 0.466 | 0.98  (0.31, 3.04) | 0.965 | 3.92  (3.19, 4.81) | <0.001 | 4.57  (3.34, 6.25) | <0.001 | 1.66  (1.41, 1.96) | <0.001 | 18.35  (8.29-40.64) | <0.001 | 8.11  (5.40-12.19) | <0.001 |
| ***S .mansoni* infection (CAA/PCR)** | | | | | | | | | | | | | | | | |
| Positive | 0.93  (0.77, 1.12) | 0.428 | 1.17  (0.87, 1.56) | 0.305 | 1.16  (0.88, 1.54) | 0.290 | 0.78  (0.61, 1.01) | 0.057 | 0.86  (0.66, 1.11) | 0.235 | 1.02  (0.83, 1.26) | 0.852 | 1.22  (0.97, 1.53) | 0.091 | 0.95  (0.85, 1.07) | 0.376 |
| Negative | Ref. |  | Ref. |  | Ref. |  | Ref. |  |  |  | Ref. |  | Ref. |  | Ref. |  |
| **Hookworm infection** | | | | | | | | | | | | | | | | |
| Positive | 1.01  (0.83, 1.24) | 0.903 | 1.03  (0.74, 1.43) | 0.863 | 1.07  (0.78, 1.47) | 0.657 | 1.07  (0.80, 1.42) | 0.663 | 0.70  (0.52, 0.93) | 0.013 | 0.75  (0.59, 0.94) | 0.015 | 1.15  (0.89, 1.48) | 0.275 | 1.12  (0.99, 1.27) | 0.073 |
| Negative | Ref. |  | Ref. |  |  |  | Ref. |  | Ref. |  | Ref. |  | Ref. |  | Ref. |  |
| ***S .mansoni* infection intensity (CAA)** | | | | | | | | | | | | | | | | |
| High | 0.72  (0.56, 0.93) | 0.043 | 1.11  (0.77, 1.61) | 0.501 | 1.10  (0.77, 1.56) | 0.518 | 0.91  (0.66, 1.25) | 0.228 | 1.20  (0.87, 1.66) | 0.400 | 1.24  (0.95, 1.61) | 0.328 | 0.94  (0.71, 1.25) | 0.426 | 0.94  (0.82, 1.09) | 0.252 |
| Medium | 1.07  (0.83, 1.38) |  | 1.05  (0.71, 1.53) |  | 1.04  (0.72, 1.49) |  | 0.71  (0.51, 0.99) |  | 0.88  (0.63, 1.22) |  | 0.94  (0.71, 1.23) |  | 0.78  (0.58, 1.05) |  | 0.86  (0.74, 0.99) |  |
| Low | 1.00  (0.79, 1.27) |  | 1.33  (0.92, 1.92) |  | 1.30  (0.92, 1.85) |  | 0.97  (0.70, 1.33) |  | 0.92  (0.67, 1.28) |  | 1.06  (0.81, 1.38) |  | 0.96  (0.72, 1.28) |  | 0.94  (0.81, 1.08) |  |
| Uninfected | Ref. |  | Ref. |  | Ref. |  | Ref. |  | Ref. |  | Ref. |  | Ref. |  | Ref. |  |
| **S. *mansoni* specific antibodies** | 0.77  (0.64, 0.93) | 0.007 | 1.01  (0.75, 1.35) | 0.973 | 1.02  (0.78, 1.35) | 0.874 | 0.87  (0.68, 1.12) | 0.290 | 0.92  (0.71, 1.19) | 0.518 | 0.99  (0.81, 1.23) | 0.980 | 0.97  (0.77, 1.21) | 0.763 | 0.98  (0.88, 1.09) | 0.705 |

GMR: Geometric mean ratio, CI: 95% confidence interval

Table 2: Crude associations of pre-vaccination parameters with vaccine responses at the second post-vaccination timepoint (48 weeks post BCG and 44 weeks post yellow fever, oral typhoid, and HPV)

|  | **BCG-specific IFN-γ** | | **Yellow fever PRNT_50_ titres** | | **Yellow fever PRNT_90_ titres** | | ***S. Typhi* O:LPS-specific IgG** | | **HPV-16-specific IgG** | | **HPV-18-specific IgG** | |
| --- | --- | --- | --- | --- | --- | --- | --- | --- | --- | --- | --- | --- |
| **Characteristic** | **GMR (95% CI)** | **P value** | **GMR (95% CI)** | **P value** | **GMR (95% CI)** | **P value** | **GMR (95% CI)** | **P value** | **GMR (95% CI)** | **P value** | **GMR (95% CI)** | **P value** |
| **Age, years** | 1.05  (0.99, 1.11) | 0.060 | 1.05  (0.97, 1.13) | 0.210 | 1.07  (0.98, 1.15) | 0.124 | 1.07  (0.99, 1.16) | 0.057 | 1.01  (0.92, 1.10) | 0.914 | 0.95  (0.88, 1.02) | 0.178 |
| **Sex (Male)** | 0.10  (0.83, 1.20) | 0.997 | 0.74  (0.57, 0.96) | 0.023 | 0.71  (0.53, 0.93) | 0.014 | 0.96  (0.74, 1.24) | 0.744 | 0.54  (0.40, 0.72) | <0.001 | 0.66  (0.52, 0.85) | 0.001 |
| **Body Mass Index (BMI)** | 1.04  (0.99, 1.09) | 0.147 | 1.03  (0.96, 1.11) | 0.397 | 1.02  (0.95, 1.10) | 0.564 | 1.08  (1.01, 1.16) | 0.027 | 0.97  (0.89, 1.06) | 0.463 | 0.95  (0.88, 1.02) | 0.137 |
| **Received immunisation as a baby before school** | | | | | | | | | | | | |
| No | 0.80  (0.58, 1.11) | 0.173 | 1.83  (1.15, 2.91) | 0.020 | 1.79  (1.09, 2.93) | 0.063 | 1.25  (0.78, 2.00) | 0.595 | 0.44  (0.26, 0.75) | 0.007 | 0.60  (0.38, 0.95) | 0.090 |
| Don’t know | 0.81  (0.61, 1.08) |  | 1.32  (0.89, 1.96) |  | 1.15  (0.76, 1.75) |  | 0.94  (0.64, 1.38) |  | 1.17  (0.75, 1.83) |  | 1.01  (0.68, 1.48) |  |
| Yes | Ref. |  | Ref. |  | Ref. |  | Ref. |  | Ref. |  | Ref. |  |
| **Received immunisation since starting school** | | | | | | | | | | | | |
| Yes | 1.19  (0.83, 1.71) | 0.556 | 0.86  (0.51, 1.46) | 0.548 | 1.19  (0.68, 2.07) | 0.814 | 1.40  (0.84, 2.32) | 0.072 | 0.92  (0.51, 1.64) | 0.911 | 1.51  (0.92, 2.48) | 0.241 |
| Don’t know | 1.12  (0.77, 1.63) |  | 1.28  (0.75, 2.18) |  | 0.96  (0.54, 1.68) |  | 1.71  (1.01, 2.90) |  | 0.90  (0.49, 1.67) |  | 0.92  (0.55, 1.55) |  |
| No | Ref. |  | Ref. |  | Ref. |  | Ref. |  | Ref. |  | Ref. |  |
| **Current Schistosoma treatment (trial arm)** | | | | | | | | | | | | |
| Treated (intensive) | 1.05  (0.88, 1.22) | 0.605 | 1.11  (0.86, 1.44) | 0.413 | 1.12  (0.86, 1.48) | 0.403 | 1.11  (0.86, 1.43) | 0.421 | 0.91  (0.68, 1.21) | 0.498 | 1.03  (0.80, 1.32) | 0.823 |
| Untreated (standard) | Ref. |  | Ref. |  | Ref. |  | Ref. |  | Ref. |  | Ref. |  |
| **Reported treatment for worms in 12 months before enrolment** | | | | | | | | | | | | |
| Yes | 1.16  (0.87, 1.56) | 0.316 | 1.09  (0.71, 1.67) | 0.704 | 1.04  (0.66, 1.63) | 0.874 | 1.11  (0.73, 1.67) | 0.636 | 1.28  (0.80, 2.06) | 0.302 | 1.26  (0.84, 1.90) | 0.259 |
| No | Ref. |  | Ref. |  | Ref. |  | Ref. |  | Ref. |  | Ref. |  |
| **Malaria infection status PCR** | 1.01  (0.79, 1.27) | 0.958 | 0.93  (0.67, 1.31) | 0.681 | 0.91  (0.64, 1.30) | 0.604 | 1.04  (0.75, 1.46) | 0.802 | 0.95  (0.65, 1.40) | 0.810 | 1.02  (0.73, 1.41) | 0.918 |
| **Reported treatment for malaria in 12 months before enrolment** | | | | | | | | | | | | |
| Yes | 1.08  (0.90, 1.30) | 0.398 | 1.15  (0.89, 1.50) | 0.292 | 1.33  (1.01, 1.76) | 0.044 | 0.95  (0.73, 1.24) | 0.710 | 0.79  (0.59, 1.07) | 0.123 | 0.99  (0.78, 1.25) | 0.903 |
| No | Ref. |  | Ref. |  | Ref. |  | Ref. |  | Ref. |  | Ref. |  |
| **Malaria specific antibodies (PfAMA-1)** | 1.08  (0.96, 1.20) | 0.196 | 1.06  (0.91, 1.24) | 0.429 | 1.00  (0.85, 1.18) | 0.975 | 0.98  (0.84, 1.14) | 0.770 | 0.85  (0.72, 1.01) | 0.068 | 0.92  (0.80, 1.07) | 0.277 |
| **Malaria specific antibodies (PfMSP-2)** | 1.15  (0.88, 1.49) | 0.310 | 0.84  (0.58, 1.22) | 0.368 | 0.80  (0.54, 1.19) | 0.276 | 1.37  (0.95, 1.96) | 0.090 | 0.88  (0.58, 1.34) | 0.554 | 0.94  (0.66, 1.35) | 0.751 |
|  |  |  |  |  |  |  |  |  |  |  |  |  |
| **Own toilet** | | | | | | | | | | | | |
| Yes | 0.99  (0.83, 1.19) | 0.939 | 1.27  (0.98, 1.64) | 0.071 | 1.19  (0.90, 1.56) | 0.221 | 1.04  (0.80, 1.33) | 0.785 | 0.89  (0.67, 1.19) | 0.430 | 0.88  (0.68, 1.12) | 0.290 |
| No | Ref. |  | Ref. |  | Ref. |  | Ref. |  | Ref. |  | Ref. |  |
| **Source of drinking water** | | | | | | | | | | | | |
| Well/piped water | 0.94  (0.78, 1.13) | 0.504 | 1.13  (0.87, 1.48) | 0.350 | 1.04  (0.79, 1.38) | 0.769 | 0.87  (0.67, 1.12) | 0.269 | 0.85  (0.64, 1.15) | 0.295 | 0.82  (0.64, 1.06) | 0.135 |
| Lake/spring | Ref. |  | Ref. |  | Ref. |  | Ref. |  | Ref. |  | Ref. |  |
| **Residence of participant at birth** | | | | | | | | | | | | |
| Village | 1.05  (0.74, 1.49) | 0.793 | 0.85  (0.50, 1.43) | 0.537 | 0.96  (0.55, 1.68) | 0.892 | 0.73  (0.44, 1.22) | 0.226 | 1.32  (0.75, 2.34) | 0.333 | 1.27  (0.78, 2.06) | 0.341 |
| Town or city | Ref. |  | Ref. |  | Ref. |  | Ref. |  | Ref. |  | Ref. |  |
| **Residence of participant between birth and age five years** | | | | | | | | | | | | |
| Village | 0.89  (0.58, 1.38) | 0.611 | 0.80  (0.43, 1.51) | 0.494 | 0.90  (0.46, 1.74) | 0.747 | 0.62  (0.34, 1.15) | 0.127 | 1.71  (0.83, 3.52) | 0.145 | 1.63  (0.88, 3.04) | 0.122 |
| Town or city | Ref. |  | Ref. |  | Ref. |  | Ref. |  | Ref. |  | Ref. |  |
| **Diet score** | 0.99  (0.97, 1.02) | 0.903 | 1.01  (0.97, 1.04) | 0.732 | 0.99  (0.96, 1.04) | 0.899 | 1.01  (0.98, 1.05) | 0.472 | 1.01  (0.97, 1.05) | 0.776 | 0.99  (0.96, 1.03) | 0.795 |
| **Pre-vaccination antigen-specific responses*** | 2.06  (1.69-2.49) | <0.001 | 1.45  (1.04, 2.02) | 0.029 | 3.19  (1.03, 9.93) | 0.045 | 5.77  (4.84, 6.89) | <0.001 | 1.43  (0.91, 2.25) | 0.121 | 1.35  (1.06, 1.71) | 0.017 |
| ***S. mansoni* infection (CAA/PCR)** | | | | | | | | | | | | |
| Positive | 0.89  (0.74, 1.08) | 0.242 | 0.89  (0.67, 1.18) | 0.423 | 1.03  (0.76, 1.38) | 0.868 | 0.73  (0.56, 0.96) | 0.025 | 1.05  (0.77, 1.44) | 0.759 | 0.88  (0.67, 1.15) | 0.348 |
| Negative | Ref. |  | Ref. |  | Ref. |  | Ref. |  |  |  | Ref. |  |
| **Hookworm infection** | | | | | | | | | | | | |
| Positive | 0.95  (0.77, 1.18) | 0.636 | 0.99  (0.72, 1.36) | 0.940 | 1.01  (0.72, 1.41) | 0.973 | 0.89  (0.66, 1.21) | 0.462 | 0.65  (0.46, 0.92) | 0.016 | 0.75  (0.55, 1.01) | 0.058 |
| Negative | Ref. |  | Ref. |  | Ref. |  | Ref. |  |  |  | Ref. |  |
| ***S .mansoni* infection intensity** | | | | | | | | | | | | |
| High | 0.67  (0.53, 0.85) | 0.011 | 0.69  (0.48, 0.98) | 0.031 | 0.82  (0.56, 1.19) | 0.128 | 0.93  (0.66, 1.32) | 0.447 | 1.46  (0.99, 2.15) | 0.054 | 1.29  (0.93, 1.79) | 0.015 |
| Medium | 0.92  (0.71, 1.19) |  | 1.07  (0.74, 1.55) |  | 1.17  (0.79, 1.73) |  | 0.80  (0.56, 1.15) |  | 0.97  (0.64, 1.48) |  | 0.69  (0.48, 0.99) |  |
| Low | 0.85  (0.67, 1.08) |  | 1.29  (0.91, 1.83) |  | 1.37  (0.94, 1.99) |  | 0.78  (0.55, 1.11) |  | 0.77  (0.52, 1.14) |  | 0.80  (0.57, 1.12) |  |
| Negative | Ref. |  | Ref. |  | Ref. |  | Ref. |  | Ref. |  | Ref. |  |
| **S. *mansoni* specific antibodies** | 0.84  (0.69, 1.01) | 0.059 | 0.60  (0.46, 0.79) | <0.001 | 0.74  (0.55, 0.99) | 0.041 | 0.84  (0.64, 1.10) | 0.207 | 1.08  (0.79, 1.47) | 0.622 | 1.05  (0.81, 1.37) | 0.712 |

IQR Interquartile range

PfMSP-2 *Plasmodium falciparum* merozoite surface protein-2

PfAMA-1 *Plasmodium falciparum* apical membrane antigen-1

Table 3: Adjusted associations of *S. mansoni* infection, *S. mansoni* infection intensity and hookworm with vaccine responses at the peak timepoint (8 weeks post BCG, 4 weeks post yellow fever, oral typhoid and HPV and 24 weeks post Tetanus and diphtheria.

|  |  |  | **BCG-specific IFN-γ** | | **Yellow fever PRNT_50_ titres** | | **Yellow fever PRNT_90_ titres** | | ***S. Typhi* O:LPS-specific IgG** | | **HPV-16-specific IgG** | | **HPV-18-specific IgG** | | **Tetanus toxoid-specific IgG** | | **Diphtheria toxoid-specific IgG** | | |
| --- | --- | --- | --- | --- | --- | --- | --- | --- | --- | --- | --- | --- | --- | --- | --- | --- | --- | --- | --- |
| **Characteristics^a^** | **Categories** |  | **GMR (95% CI)** | **P**  **value^*^** | **GMR (95% CI)** | **P**  **value^*^** | **GMR (95% CI)** | **P value** | **GMR**  **(95% CI)** | **P value** | **GMR**  **(95% CI)** | **P**  **value^*^** | **GMR (95% CI)** | **P**  **value^*^** | **GMR**  **(95% CI)** | **P**  **value^*^** | **GMR (95% CI)** | **P**  **value*** |  |
| ***S. mansoni* infection status** | | | | | | | | | | | | | | | | | | |  |
| *S. mansoni* infection at baseline (CAA and PCR) | Infected |  | 1.15  (0.89, 1.50) | 0.288 | 1.27  (0.89, 1.80) | 0.181 | 1.29  (0.92, 1.80) | 0.134 | 0.67  (0.52, 0.86) | 0.002 | 0.91  (0.71, 1.17) | 0.450 | 0.98  (0.78, 1.22) | 0.825 | 1.21  (0.95, 1.55) | 0.126 | 1.06  (0.94, 1.20) | 0.332 |  |
|  | Uninfected |  | Ref. |  | Ref. |  | Ref. |  | Ref. |  | Ref. |  | Ref. |  | Ref. |  | Ref. |  |  |
| Pre-vaccination immune responses |  |  | 1.56  (1.23, 1.98) | <0.001 |  |  |  |  | 3.86  (3.06, 4.86) | <0.001 | 3.65  (2.57, 5.18) | <0.001 | 1.51  (1.25, 1.82) | <0.001 | 14.78  (6.08, 35.88) | <0.001 | 8.34  (5.30, 13.14) | <0.001 |  |
| Received immunisation as a baby before school | Yes |  | 1.78  (1.16, 2.73) | 0.021 |  |  |  |  |  |  |  |  |  |  | 1.65  (1.13, 2.44) | 0.038 |  |  |  |
|  | Don’t know |  | 1.44  (0.86, 2.41) |  |  |  |  |  |  |  |  |  |  |  | 1.53  (0.94, 2.47) |  |  |  |  |
|  | No |  | Ref. |  |  |  |  |  |  |  |  |  |  |  | Ref. |  |  |  |  |
| Received immunisation since starting school | Yes |  |  |  | 0.43  (0.23, 0.80) | 0.008 | 0.37  (0.20, 0.67) | 0.005 |  |  | 2.44  (1.54, 3.85) | <0.001 | 2.43  (1.64, 3.59) | <0.001 |  |  |  |  |  |
|  | Don’t know |  |  |  | 0.77  (0.38, 1.56) |  | 0.82  (0.42, 1.60) |  |  |  | 0.91  (0.55, 1.50) |  | 0.94  (0.61, 1.44) |  |  |  |  |  |  |
|  | No |  |  |  | Ref. |  | Ref. |  |  |  | Ref. |  | Ref. |  |  |  |  |  |  |
| Owns toilet | Yes |  |  |  | 1.39  (1.02, 1.90) | 0.038 | 1.46  (1.09, 1.98) | 0.013 |  |  |  |  |  |  |  |  |  |  |  |
| Sex | Male |  |  |  |  |  |  |  |  |  | 0.60  (0.48, 0.75) | <0.001 | 0.66  (0.54, 0.80) | <0.001 |  |  |  |  |  |
| Malaria positive |  |  | 1.49  (1.08, 2.04) | 0.015 | 0.55  (0.35, 0.86) | 0.009 | 0.59  (0.38, 0.90) | 0.014 |  |  |  |  |  |  |  |  |  |  |  |
| ***S. mansoni* infection intensity** | | | | | | | | | | | | | | | | | | |  |
|  | High | Intensive  Standard | 1.36  (0.87, 2.12)  0.70  (0.45, 1.09) | 0.004 | 1.11  (0.72, 1.71) | 0.433 | 1.06  (0.70, 1.61) | 0.589 | 0.72  (0.52, 0.99) | 0.084 | 1.19  (0.87, 1.63) | 0.595 | 1.26  (0.96, 1.65) | 0.375 | 1.01  (0.74, 1.38) | 0.568 | 1.09  (0.94, 1.27) | 0.162 |  |
|  | Medium | Intensive  Standard | 0.80  (0.48, 1.34)  1.5  (0.99, 2.40) |  | 1.08  (0.70, 1.66) |  | 1.08  (0.72, 1.64) |  | 0.70  (0.51, 0.97) |  | 0.94  (0.68, 1.28) |  | 1.02  (0.78, 1.33) |  | 0.82  (0.61, 1.11) |  | 0.91  (0.79, 1.06) |  |  |
|  | Low | Intensive  Standard | 1.49  (0.94, 2.36)  0.91  (0.58, 1.42) |  | 1.42  (0.94, 2.15) |  | 1.32  (0.89, 1.96) |  | 0.88  (0.65, 1.20) |  | 1.01  (0.75, 1.37) |  | 1.08  (0.83, 1.40) |  | 0.92  (0.67, 1.25) |  | 1.07  (0.92, 1.24) |  |  |
|  | Negative |  | Ref. |  | Ref. |  | Ref. |  | Ref. |  | Ref. |  | Ref. |  | Ref. |  | Ref. |  |  |
|  |  |  |  |  |  |  |  |  |  |  |  |  |  |  |  |  |  |  |  |
| **Hookworm** | Infected |  | 0.94  (0.72, 1.23) | 0.669 | 1.03  (0.71, 1.51) | 0.875 | 1.08  (0.75, 1.55) | 0.672 | 1.22  (0.92, 1.62) | 0.170 | 0.66  (0.51, 0.86) | 0.003 | 0.82  (0.65, 1.04) | 0.099 | 1.26  (0.97, 1.63) | 0.085 | 1.16  (1.02, 1.31) | 0.023 |  |
|  | Uninfected |  | Ref. |  | Ref. |  | Ref. |  | Ref. |  | Ref. |  | Ref. |  | Ref. |  | Ref. |  |  |
|  |  |  |  |  |  |  |  |  |  |  |  |  |  |  |  |  |  |  |  |

*^a^* Separate models were fit for each exposure of interest i.e. *S. mansoni* infection*, S. mansoni* intensity and hookworm infections

**S. mansoni* infection results adjusted for age, sex, BMI, prior immunisation, prior treatment of worms/malaria, malaria infection residence at birth and between birth and five years, diet score

Table 4: Adjusted associations of *S. mansoni*, *S. mansoni* infection intensity and hookworm infection with vaccine responses at the waning timepoint (48 weeks post BCG and 44 weeks post yellow fever, oral typhoid, and HPV)

| **Characteristics^a^** | **Categories** | **BCG-specific IFN-γ** | | **Yellow fever PRNT_50_ titres** | | **Yellow fever PRNT_90_ titres** | | ***S. Typhi* O:LPS-specific IgG** | | **HPV-16-specific IgG** | | **HPV-18-specific IgG** | |
| --- | --- | --- | --- | --- | --- | --- | --- | --- | --- | --- | --- | --- | --- |
|  |  | **GMR (95% CI)** | **P value^*^** | **GMR (95% CI)** | **P value^*^** | **GMR (95% CI)** | **P value^*^** | **GMR (95% CI)** | **P value^*^** | **GMR (95% CI)** | **P value^*^** | **GMR (95% CI)** | **P value^*^** |
| ***S. mansoni* infection status** |  |  |  |  |  |  |  |  |  |  |  |  |  |
| *S. mansoni* infection at baseline (CAA and PCR) | Infected | 0.85  (0.66, 1.10) | 0.223 | 1.01  (0.73, 1.40) | 0.968 | 1.08  (0.76, 1.54) | 0.653 | 0.74  (0.60, 0.92) | 0.007 | 0.99  (0.68, 1.46) | 0.990 | 0.78  (0.57, 1.06) | 0.106 |
|  | Uninfected | Ref. |  | Ref. |  | Ref. |  | Ref. |  | Ref. |  | Ref. |  |
| Pre-vaccination immune responses |  | 1.96  (1.55, 2.47) | <0.001 |  |  |  |  | 5.79  (4.74, 7.06) | <0.001 |  |  | 1.37  (1.03, 1.82) | 0.031 |
| Received immunisation as a baby before school | Yes |  |  | 0.57  (0.35, 0.94) | 0.042 |  |  |  |  | 2.42  (1.37, 4.29) | 0.007 | 1.81  (1.14, 2.88) | 0.041 |
|  | Don’t know |  |  | 0.81  (0.44, 1.50) |  |  |  |  |  | 2.79  (1.35, 5.76) |  | 1.83  (1.01, 3.31) |  |
|  | No |  |  | Ref. |  |  |  |  |  | Ref. |  | Ref. |  |
|  |  |  |  |  |  |  |  |  |  |  |  |  |  |
| Treated for worms in the last 12 months | Yes | 1.61  (1.05, 2.48) | 0.029 |  |  |  |  |  |  |  |  |  |  |
| Sex | Male |  |  | 0.73  (0.54, 0.99) | 0.047 | 0.69  (0.50, 0.96) | 0.026 |  |  | 0.57  (0.40, 0.82) | 0.002 |  |  |
| Drinking water | (well/piped) |  |  |  |  |  |  |  |  |  |  | 0.69  (0.51, 0.92) | 0.013 |
| Age |  |  |  |  |  | 1.13  (1.01, 1.27) | 0.035 |  |  |  |  |  |  |
| ***S. mansoni* infection intensity** | | | | | | | | | | | | | |
|  | High | 0.70  (0.50, 0.98) | 0.080 | 0.79  (0.52, 1.22) | 0.205 | 0.92  (0.58, 1.45) | 0.407 | 0.83  (0.62, 1.09) | 0.122 | 1.47  (0.91, 2.39) | 0.234 | 1.21  (0.82, 1.77) | 0.037 |
|  | Medium | 0.86  (0.61, 1.20) |  | 1.06  (0.70, 1.61) |  | 1.12  (0.71, 1.75) |  | 0.73  (0.55, 0.95) |  | 0.98  (0.61, 1.57) |  | 0.64  (0.44, 0.94) |  |
|  | Low | 0.73  (0.53, 0.99) |  | 1.35  (0.90, 2.03) |  | 1.38  (0.90, 2.13) |  | 0.91  (0.69, 1.19) |  | 0.83  (0.52, 1.32) |  | 0.89  (0.62, 1.29) |  |
|  | Negative | Ref. |  | Ref. |  | Ref. |  | Ref. |  | Ref. |  | Ref. |  |
|  |  |  |  |  |  |  |  |  |  |  |  |  |  |
| **Hookworm** | Infected | 0.89  (0.68, 1.16) | 0.375 | 1.02  (0.72, 1.46) | 0.902 | 1.05  (0.72, 1.53) | 0.814 | 0.99  (0.80, 1.24) | 0.966 | 0.77  (0.51, 1.16) | 0.212 | 0.82  (0.59, 1.13) | 0.226 |
|  | Uninfected | Ref. |  | Ref. |  | Ref. |  | Ref. |  | Ref. |  | Ref. |  |
|  |  |  |  |  |  |  |  |  |  |  |  |  |  |

*^a^* Separate models were fit for each exposure of interest i.e. *S. mansoni* infection*, S. mansoni* intensity and hookworm infections

* *S. mansoni* infection results adjusted for age, sex, BMI, prior immunisation, prior treatment of worms/malaria, malaria infection, residence at birth and between birth and five years, diet score

Table 5: Adjusted associations of *S. mansoni* intensity with vaccine responses

| **Infection intensity** | **Adjusted GMR(95%) CI** | **P value** |
| --- | --- | --- |
| **BCG-specific IFN-γ** |  |  |
| High versus negative |  |  |
| Intensive | 0.93(0.67, 1.29) | 0.669 |
| Standard | 0.65(0.46, 0.91) | 0.013 |
| Medium versus negative |  |  |
| Intensive | 0.77(0.52, 1.12) | 0.168 |
| Standard | 1.32(0.93, 1.87) | 0.125 |
| Low versus negative |  |  |
| Intensive | 0.96(0.68, 1.35) | 0.800 |
| Standard | 0.86(0.60, 1.22) | 0.392 |
|  |  |  |
| **Yellow fever PRNT_50_ titres** |  |  |
| High versus negative | 0.98(0.72, 1.32) | 0.871 |
| medium versus negative | 1.07(0.79, 1.45) | 0.644 |
| Low versus negative | 1.39(1.04, 1.85) | 0.027 |
|  |  |  |
| **Yellow fever PRNT_90_ titres** |  |  |
| High versus negative | 1.01(0.74, 1.36) | 0.961 |
| medium versus negative | 1.11(0.82, 1.49) | 0.514 |
| Low versus negative | 1.35(1.01, 1.80) | 0.044 |
|  |  |  |
| ***S. Typhi* O:LPS-specific IgG** |  |  |
| High versus negative | 0.74(0.59, 0.94) | 0.013 |
| medium versus negative | 0.70(0.55, 0.88) | 0.002 |
| Low versus negative | 0.87(0.69, 1.09) | 0.215 |
|  |  |  |
| **HPV-16-specific IgG** |  |  |
| High versus negative | 1.25(0.93, 1.66) | 0.137 |
| medium versus negative | 0.93(0.70, 1.24) | 0.607 |
| Low versus negative | 0.94(0.71, 1.23) | 0.633 |
|  |  |  |
| **HPV-18-specific IgG** |  |  |
| High versus negative | 1.22(0.96, 1.54) | 0.108 |
| medium versus negative | 0.82(0.65, 1.04) | 0.095 |
| Low versus negative | 0.99(0.79, 1.25) | 0.977 |

*Supplementary figures*

Figure S1: Vaccine responses by *Schistosoma mansoni* infection status (positive versus negative). Shown are unadjusted responses at pre-vaccination (week 0 for BCG, yellow fever, oral typhoid and HPV), (week 28 for tetanus and diphtheria). Post vaccination responses are shown at (weeks 8 and 52) timepoints. Plots show individual data points, a horizontal line and whiskers denoting the geometric mean and 95% CI, respectively. **SFUs**: ELISpot assay spot forming units; **PBMCs**: peripheral blood mononuclear cells; **PRNT_50_**: plaque reduction neutralizing reference tests at 50% neutralization; **HPV-16**: Human Papillomavirus type 16; **HPV-18**: Human Papillomavirus type 18; **TT**: tetanus toxoid.

Figure S2: Vaccine responses by *Schistosoma mansoni* infection intensity. Shown are unadjusted responses at pre-vaccination (week 0 for BCG, yellow fever, oral typhoid and HPV), (week 28 for tetanus and diphtheria). Post vaccination responses are shown at (weeks 8 and 52) timepoints. Plots show individual data points, a horizontal line and whiskers denoting the geometric mean and 95% CI, respectively. **SFUs**: ELISpot assay spot forming units; **PBMCs**: peripheral blood mononuclear cells; **PRNT_50_**: plaque reduction neutralizing reference tests at 50% neutralization; **HPV-16**: Human Papillomavirus type 16; **HPV-18**: Human Papillomavirus type 18; **TT**: tetanus toxoid; **DT**: diphtheria toxoid.

Figure S3: Association of *S. mansoni* infection status (positive versus negative) before vaccination with post-vaccination response for BCG, yellow fever, oral typhoid and HPV. Shown are predicted responses from a linear mixed model. **SFUs**: ELISpot assay spot forming units; **PBMCs**: peripheral blood mononuclear cells; **PRNT_50_**: plaque reduction neutralizing reference tests at 50% neutralization; **HPV-16**: Human Papillomavirus type 16; **HPV-18**: Human Papillomavirus type 18.

Figure S4: Association of pre-vaccination hookworm infection status (positive versus negative) with post-vaccination response for BCG, yellow fever, oral typhoid and HPV. Shown are predicted responses from a linear mixed model. **SFUs**: ELISpot assay spot forming units; **PBMCs**: peripheral blood mononuclear cells; **PRNT_50_**: plaque reduction neutralizing reference tests at 50% neutralization; **HPV-16**: Human Papillomavirus type 16; **HPV-18**: Human Papillomavirus type 18.

*Supplementary methods*

***Ex vivo* interferon-γ ELISpot assays to quantify BCG-specific responses**

To quantify BCG-specific responses, we conducted *ex vivo* interferon (IFN)-γ ELISpot assays, using a Human IFN-γ (ALP) ELISpot Flex kit (Mabtech, Sweden) and multiscreen-IP 0.45μm filter 96-well plates (Merck Millipore). ELISpot plates were coated overnight at 4°C with 50 μl of anti-IFN-γ capture antibody (15 μg/ml) dissolved in 0.05 M carbonate-bicarbonate buffer (Sigma Aldrich). The plates were then washed 5 times with sterile 1X PBS (Sigma Aldrich), and blocked (2-5 hours, 37°C) by adding 100 μl/well of R10 medium (10% fetal bovine serum [Sigma Adrich] in RPMI 1640 medium [Thermofisher scientific] supplemented with L-glutamine, streptomycin, HEPES buffer and penicillin [all from Life technologies, UK]). During plate blocking, peripheral blood mononuclear cells (PBMCs) were isolated from heparinised whole blood by density gradient centrifugation with Histopaque® (Sigma Aldrich). For each study sample, PBMCs (300,000 per test well) were stimulated in duplicate for 18-20 hours at 37^o^C, 5% CO_2_, with BCG (Moscow strain, Serum Institute of India) at a concentration of 200,000 colony forming units per ml, or left unstimulated. Staphylococcal enterotoxin B (SEB; Sigma Aldrich) was used at a final concentration of 10 μg/ml as a positive control, and a 1:1 mix of the 6-kDa early secretory antigenic target and 10-kDa culture filtrate protein (ESAT-6 and CFP-10 recombinant proteins from BEI Resources, USA) used at a final concentration of 2.5 μg/ml for exploratory assessment of tuberculosis infection. Following the 18-20 hour incubation, plates were washed 5 times with 200ul/well PBS containing 0.05% Tween 20 (Sigma Aldrich) and incubated for 2 hours at room temperature with 50 μl per well of a 1/1000 PBS dilution of biotin anti-IFN-γ antibody from the ELISpot kit. After another washing step with PBS-0.05% Tween 20, plates were incubated for 1 hour at room temperature with 50 μl per well of a 1/1000 PBS dilution of a streptavidin-alkaline phosphatase conjugate from the ELISpot kit. Plates were washed, developed for 3-10 minutes with 50 μl per well of 5-Bromo-4-chloro-3-indoxyl phosphate/Nitro blue tetrazolium (BCIP/NBT; Europa Bioproducts), and the reaction stopped by washing the plate under tap water. Plates were allowed to dry in the dark at room temperature and read using an ELISpot reader (Autoimmun Diagnostika Gmbh iSpot , Strassberg, Germany) running AID ELISpot software v.7.0. Spot-forming units (SFUs) per well were manually verified to remove artefacts.

We performed QC through a number of steps: 1) for each sample, we checked to ascertain whether the PBMC isolation procedure was conducted within eight hours after sample collection; 2) we inspected each ELISpot plate visually for quality and completeness of labelling (sample IDs, date, time point and antigens) and compared the plate picture to the exported spot count spreadsheet to ensure the correct data had been exported; 3) we checked the calculated data in the exported database to ensure the background subtraction, average of duplicate wells and multiplication up to spot forming units per million PBMC had been performed correctly; 4) we assessed whether the unstimulated well and SEB well controls for each assay were within the accepted range.

Results were reported as SFUs per a million PBMCs, calculated sequentially by 1) subtracting mean SFUs of unstimulated wells from mean SFUs of duplicate antigen wells, and 2) correcting for the number of PBMCs per well (300,000). Samples that had more than 83.3 SFUs per a million PBMCs in the unstimulated well were considered invalid and not included in the final analysis.

**Yellow Fever plaque reduction neutralizing reference test (PRNT)**

A plaque reduction assay as described by Beaty *et al* (1). was used. Briefly, Vero cells at a concentration of 65,000 cells/ml were seeded into 6-well plates (Greiner Bio-One GmbH, Germany) at a volume of 3 ml/well. Cells were cultured in growth medium (1X Eagle’s Minimum Essential Medium, 8% heat inactivated fetal bovine serum, 100 units penicillin/streptomycin, gentamycin 50 mg/ml and fungizone 1 mg/ml) at 37^o^C (with 5% CO_2_) for 3–4 days. Culture medium was then removed from the cell monolayer by dumping. Test plasma were inactivated at 56^o^C for 30 min to remove complement factor, serially (two-fold) diluted from 1:10 to 1:20480 in BA-1 diluent (10X M199 Hanks’ Salts without L-Glutamine, 5% Bovine Serum Albumin, 1M TRIS-HCL pH 7.5, L-Glutamine, sodium bicarbonate 7.5%, 100X penicillin/streptomycin, 1000X fungizone in sterile water), and mixed with approximately 200 Plaque Forming Units (PFU) of a reference YF-17D virus preparation. The plasma-virus mixture (0.1 ml) was added to the confluent monolayer of Vero cells in each well and incubated at 37^o^C (with 5% CO_2_) for 1 hour. The first overlay medium (comprising Miller’s 2X Yeast Extract-Lactalbumin hydrolysate medium, 10X Earle’s Buffered Salts Solutions, 2% fetal bovine serum, 1000X fungizone, 1000X Gentamycin, and 2% low-melting agarose) was added, 3 ml per well, and allowed to solidify for 30 minutes at room temperature. The plates were incubated at 37^o^C with 5% CO_2_ for 4 days. To stain cell layers, a Neutral Red dye (Sigma Aldrich) second overlay was added, 2 ml per well, and allowed to solidify for 30 minutes at room temperature. After this second overlay, plates were incubated at 37^o^C in 5% CO_2_ for 2 days: plaques were counted first on day 1 and the final score documented on day 2 to establish 50% and 90% neutralization titres. Back titration plates were established to ensure infectivity of cell monolayer and standardization of virus to 200 PFU/0.1 ml. Neutralisation titres <1:10 were considered negative. Titres of 1:10 were interpreted as borderline. The PRNT antibody titres presented refer to the reciprocal of the last plasma dilution that reduced by 50% (PRNT50) or 90% (PRNT90) the number of virus plaque clusters infected by 100 PFU/0.1 ml of the reference 17D virus preparation.

For quality control, we used a high titre positive control (PC), with the last six titre dilution range, from 640 to 20480. So long as the PC titre was within the expected range and did not vary by greater than 4-fold, the assay passed quality control. Furthermore, we ran back-titrations of the virus inoculum (standardised to 200 PFU/0.1 ml) to determine the end-point specimen antibody titre at 50% or 90% neutralisation. The number of virus plaques infected at 50% neutralisation and at 90% neutralisation were expected to be within an approximate range of 25-100 and 5-20, respectively.

**Detection of plasma IgG against *Salmonella* Typhi O-lipopolysaccharide (O:LPS)**

Specific IgG to S. Typhi O-lipopolysaccharide (O:LPS) was measured by an in-house ELISA. Nunc Maxisorp 96-well plates (Thermo Fisher) were coated overnight at 4ºC with 50μl/well of 10 μg/ml of *S.* Typhi O:LPS (Sigma L2387) in bicarbonate (Na_2_CO_3_ + NaHCO_3_) buffer (0.1M, pH 9.6). Plates were washed with phosphate-buffered saline (PBS 1X)-Tween 20 (0.05%) solution, blocked with 200μl of 5% skimmed milk diluted in PBS-Tween 20 for 1 hour at room temperature (RT), washed again and incubated for 2 hours at RT with 50μl of test plasma samples (diluted 1/320 with 1% skimmed milk in PBS-Tween 20) and two-fold serially diluted standard sera (top concentration 20 EU/ml). Standards were derived from a pooled sample generated from sera of known O:LPS-specific IgG titres, kindly provided by the Oxford Vaccine Centre Biobank. These sera had been collected from the highest responders to O-antigen following challenge with *S.* Typhi in a controlled human infection study (2). Following test and standard sample incubation, plates were washed and O:LPS-specific IgG binding detected by incubating the plates for 1 hour at RT with goat anti-human IgG-horseradish peroxidase conjugate (Insight Biotechnology, UK), diluted 1/6000 in 1% skimmed milk–PBS-Tween 20. Plates were washed and developed by addition of 100μl of o-phenylenediamine (Sigma-Aldrich) and reactions stopped after 5 minutes with 30μl of 2M Sulphuric acid. Optical density (OD) values were measured at 490nm (reference wavelength 630nm) on a 96-well plate ELISA reader (BioTek ELx808, USA). Nominal ELISA units (EU/ml), representing O:LPS-specific IgG titres, were interpolated from standard curves using a five-parameter curve fit using Gen5 data collection and analysis software (BioTek Instruments Inc, Vermont, Winooski, USA).

## **Detection of plasma IgG against Human Papillomavirus type 16 (HPV-16) and HPV-18**

Anti-HPV-16 and HPV-18 IgG concentrations were measured by ELISA, as previously described (3-6). Nunc Maxisorp 96-well plates (Thermo Fisher) were coated with 100 µl of HPV-16 L1 virus-like particles (VLP) at a concentration of 2.7 µg/ml, or with HPV-18 L1-L2 VLP at a concentration of 2 µg/ml and incubated at 4°C overnight. Plates were washed three times with a 1X phosphate-buffered saline (PBS)-Tween 20 (0.25%) solution, and blocked for 90 minutes at room temperature (RT) with 4% skimmed milk diluted in a 1X PBS-0.25% Tween 20 solution. The plates were washed three times and and incubated (with gentle shaking) for 1 hour at RT with 100 μl of test plasma samples, assay controls and two-fold serially diluted standard sera. Pre-vaccination test plasma samples were diluted 1/50 (HPV-16 assay) or 1/200 (HPV-18 assay) with blocking buffer, while post-vaccination plasma samples were diluted 1/400 for both HPV-16 and HPV-18 assays. Standard sera were used at a top concentration of 1.28 EU/ml and 8.2425 EU/ml for HPV-16 and HPV-18 assays, respectively. Following test and standard sample incubation, plates were washed four times and further incubated for 1 hour at RT with peroxidase-labeled goat anti-human IgG (KPL, Gaithersburg, Maryland). Plates were then developed with a tetramethylbenzidine substrate solution (KPL, Inc.) for 25 minutes in the dark at room temperature. Next, the reaction was stopped by adding 100 μl of 0.36N H_2_SO_4_ to each well. Optical density (OD) values were measured at 450 nm (reference wavelength 630 nm) on a 96-well plate ELISA reader (BioTek ELx808, USA). Nominal ELISA units (EU/ml), representing HPV-16 L1 VLP- and HPV-18 L1-L2 VLP-specific IgG titres, were interpolated from standard curves using a five-parameter curve fit using Gen5 data collection and analysis software (BioTek Instruments Inc, Vermont, Winooski, USA).

For quality control (QC), the acceptable R^2^ for the standard curve was ≥ 0.990 and the average optical density (OD) range of the top standard was 2.0-4.0. The acceptable OD of the last (8^th^) standard dilution was ≤0.25. The percentage difference in ODs between standard dilutions (i.e. n and n+1 dilution) was expected to be ≥ 0.3. Plates were repeated if they failed to meet these standard curve criteria. The calculated negative control cut-off was 4±3 EU/ml and 60±10 EU/ml for HPV-16 and HPV-18, respectively. Plates whose negative control concentration was above the cut-offs were repeated. The calculated positive control concentration was 450±10 EU/ml and 4500±10 EU/ml for HPV-16 and HPV-18 respectively. Background signal was measured by a blank whose OD was expected to be ≤0.05. Higher ODs indicated assay contamination and plates were repeated.

**ELISA measurement of anti-diphtheria and anti-tetanus IgG**

Nunc Maxisorp 96-well plates (Thermo Fisher) were coated with 50 μl of either 2 Lf units per ml of diphtheria toxoid (NIBSC product code 13/212) per ml or 0.56 Lf units per ml of tetanus toxoid (NIBSC product code 02/232) in Na_2_CO_3_/NaHCO_3_ buffer (0.1M, pH 9.6) overnight at 4°C. Plates were washed with 0.05% Tween 20 in 1X phosphate-buffered saline (PBST) and blocked for 1 hour with 5% skimmed milk powder in PBST at 37 °C. The plates were washed four times and and incubated for 2 hours at 37 °C with 50 μl of test plasma samples, and serially diluted WHO International Standard anti-toxins for diphtheria (NIBSC 10/262) or tetanus (NIBSC 13/240). Samples and standards were prepared in PBST + 1% skimmed milk (assay buffer). Pre-vaccination test samples were added at a dilution of 1/150, while post-vaccination plasma samples were added at a dilution of 1/300 in assay buffer. The standards were used at a top concentration of 3 IU/ml and 0.125 IU/ml for the tetanus and diphtheria assays, respectively. Plates were washed four times and incubated for 1 hour at 37 °C with 50 μl of polyclonal rabbit anti-human IgG HRP-conjugate (Agilent Dako, CA, USA) diluted 1/3000 in assay buffer. After another washing step, plates developed by adding 100 μl/well of o-phenylenediamine (Sigma-Aldrich) and reactions stopped after 5 minutes with 25 μl/well of 2M sulphuric acid. Optical density (OD) values were measured at 490nm (reference wavelength 630nm) on a 96-well plate ELISA reader (BioTek ELx808, USA). Tetanus and diphtheria toxoid-specific IgG concentrations (IU/ml) were interpolated from standard curves using a five-parameter curve fit using Gen5 data collection and analysis software (BioTek Instruments Inc, Vermont, Winooski, USA).

**Detection of *Plasmodium falciparum* DNA by PCR**

Prior to the PCR, DNA extraction was performed using the QIAamp DNA Blood Mini Kit (Catalogue number 51106, QIAGEN). Briefly, whole blood pellet samples stored at -80^0^C were retrieved and thawed at room temperature. Reagents in the extraction kit were reconstituted as per the manufacturer’s instructions. Volumes of retrieved samples were checked and Phosphate Buffered Saline (PBS) was added to samples with inadequate volumes. These were vortexed thoroughly for 2 to 3 minutes. The protease enzyme (20 µl) was pipetted to the bottom of a 2 ml Eppendorf tube, and the whole blood pellet (200 µl) was transferred to the tube. The AL lysis buffer (200 µl), after thorough mixing, was added and pulse vortexed for 20 seconds. The samples were then placed in a heating block set to 56^0^C for 15 minutes and thereafter centrifuged at 6000g for 1 minute to ensure no sample was trapped in the Eppendorf tube lid. 200 µl of absolute molecular grade ethanol was added to the sample, pulse vortexed, and then centrifuged. 500 µl of the sample was transferred to a spin column and centrifuged at 6000g for 1 minute, discarding the flow-through thereafter. This process was repeated for the remainder of the sample. The DNA was then purified using consecutive washes with AW1 (500ul, 250ul) and AW2 (500ul) buffers. The QIAGEN AE buffer (150 μl) was used to elute the DNA. The samples were then stored at -20^0^C until used in the PCR.

The real-time PCR was performed with the ABI 7500 Fast Real-time machine and data processed using 7500 Fast Systems software version 1.5.1. The PCR reaction was performed with a final volume of 25 μl containing 2 μl of DNA 1 μl of PhHV DNA (as an internal control, detailed below) and 22 μl of PCR master mix made of HotStarTaq Master Mix (Catalogue Number 203446), primers, and probes. The primers and probes used in this study are listed below.

PFal-F 5`-CCG ACT AGG TGT TGG ATG AAA GTG TTA A-3`

Plas-171R 5`-AAC CCA AAG ACT TTG ATT TCT CAT AA-3

Pfal114-XS_YY 5`-CTT TCG AGG TGA CTT TTA GAT-3`-BHQ1

Phocine herpes virus (PhHV) DNA, extracted from the Phocine herpes virus (kindly provided by Dr. Martin Schutten, Erasmus Medical Center, Rotterdam, the Netherlands), was included in the PCR master mix, thus distributed to all reaction wells as an internal control to check for PCR inhibition. The PhHV forward primer PhHV-267s (5’-GGGCGAATCACAGATTGAATC-3’), reverse primer PhHV-337as (5’-GCGGTTCCAAACGTACCAA-3’) and probe PhHV-305tq (Cy5-5’-TTTTTATGTGTCCGCCACCATCTGGATC-3’-BHQ2) were used for Phocin herpes virus DNA detection. A pool of DNA extracted from *P. falciparum* positive samples was used to set serially diluted standards tested alongside the samples on every plate run. The amplification conditions were 15 minutes at 95^o^C, 50 cycles of 15 seconds at 95^o^C, 30s at 60^o^C, and 30s at 72^o^C.

**Supplementary References**

1. Beaty BJ, Calisher CH, Shope RE. Arboviruses. In: Schmidt NJ, Lennette DA, Lennette ET, Lennette EH, Emmons RW, eds. Diagnostic Procedures for Viral, Rickettsial and Chlamydial Infections. 7th ed. Washington DC: American Public Health Association; 1995: 204-5.

2. Gibani MM, Jin C, Shrestha S, et al. Homologous and heterologous re-challenge with Salmonella typhi and Salmonella paratyphi A in a randomised controlled human infection model. *PLoS neglected tropical diseases* 2020; **14**(10): e0008783.

3. Dauner JG, Pan Y, Hildesheim A, Kemp TJ, Porras C, Pinto LA. Development and application of a GuHCl-modified ELISA to measure the avidity of anti-HPV L1 VLP antibodies in vaccinated individuals. *Mol Cell Probes* 2012; **26**(2): 73-80.

4. Dauner JG, Pan Y, Hildesheim A, Harro C, Pinto LA. Characterization of the HPV-specific memory B cell and systemic antibody responses in women receiving an unadjuvanted HPV16 L1 VLP vaccine. *Vaccine* 2010; **28**(33): 5407-13.

5. Pinto LA, Kemp TJ, Torres BN, et al. Quadrivalent Human Papillomavirus (HPV) Vaccine Induces HPV-Specific Antibodies in the Oral Cavity: Results From the Mid-Adult Male Vaccine Trial. *The Journal of infectious diseases* 2016; **214**(8): 1276-83.

6. Miller CN, Kemp TJ, Abrahamsen M, et al. Increases in HPV-16/18 antibody avidity and HPV-specific memory B-cell response in mid-adult aged men post-dose three of the quadrivalent HPV vaccine. *Vaccine* 2021; **39**(37): 5295-301.
